# DNA methylation, combined with RNA sequencing, provide novel insight into molecular classification of chordomas and their microenvironment

**DOI:** 10.1186/s40478-023-01610-0

**Published:** 2023-07-11

**Authors:** Szymon Baluszek, Paulina Kober, Natalia Rusetska, Michał Wągrodzki, Tomasz Mandat, Jacek Kunicki, Mateusz Bujko

**Affiliations:** 1 Department of Molecular and Translational Oncology, Maria Sklodowska-Curie National Research Institute of Oncology, Warsaw, Poland; 2 Department of Experimental Immunotherapy, Maria Sklodowska-Curie National Research Institute of Oncology, Warsaw, Poland; 3 Department of Cancer Pathomorphology, Maria Sklodowska-Curie National Research Institute of Oncology, Warsaw, Poland; 4 Department of Neurosurgery, Maria Sklodowska-Curie National Research Institute of Oncology, Warsaw, Poland

**Keywords:** Skull base chordoma, DNA methylation, Epigenetic, Gene expression, *TBXT*, Copy number variation

## Abstract

Chordomas are rare tumors of notochord remnants, occurring mainly in the sacrum and skull base. Despite of their unusually slow growth, chordomas are highly invasive and the involvement of adjacent critical structures causes treatment challenges. Due to the low incidence, the molecular pathogenesis of this entity remains largely unknown. This study aimed to investigate DNA methylation abnormalities and their impact on gene expression profiles in skull base chordomas. 32 tumor and 4 normal nucleus pulposus samples were subjected to DNA methylation and gene expression profiling with methylation microarrays and RNA sequencing. Genome-wide DNA methylation analysis revealed two distinct clusters for chordoma (termed subtypes C and I) with different patterns of aberrant DNA methylation. C Chordomas were characterized by general hypomethylation with hypermethylation of CpG islands, while I chordomas were generally hypermethylated. These differences were reflected by distinct distribution of differentially methylated probes (DMPs). Differentially methylated regions (DMRs) were identified, indicating aberrant methylation in known tumor-related genes in booth chordoma subtypes and regions encoding small RNAs in subtype C chordomas. Correlation between methylation and expression was observed in a minority of genes. Upregulation of *TBXT* in chordomas appeared to be related to lower methylation of tumor-specific DMR in gene promoter. Gene expression-based clusters of tumor samples did not overlap with DNA methylation-based subtypes. Nevertheless, they differ in transcriptomic profile that shows immune infiltration in I chordomas and up-regulation of cell cycle in C chordomas. Immune enrichment in chordomas I was confirmed with 3 independent deconvolution methods and immunohistochemistry. Copy number analysis showed higher chromosomal instability in C chordomas. Nine out of eight had deletion of *CDKN2A*/*B* loci and downregulation of genes encoded in related chromosomal band. No significant difference in patients’ survival was observed between tumor subtypes, however, shorter survival was observed in patients with higher number of copy number alterations.

**Supplementary Information:**

The online version contains supplementary material available at 10.1186/s40478-023-01610-0.

## Introduction

Chordomas are rare bone tumors originating from remnants of the notochord [1]. They most commonly occur in the sacral spine (constituting approximately 50% of cases) and in spheno-occipital region (skull base chordomas, about 30% of cases), and the remainder is distributed along the entire length of the spine. These tumors are diagnosed twice as often in men than in women [2]. Surgery remains the main modality of treatment of patients with skull base chordomas (usually via transnasal endoscopy) while radiotherapy is often used as adjuvant treatment [3]. Chordomas grow slowly, but are highly invasive. Because of the location and growth pattern, complete surgical removal is commonly unfeasible and relatively high resistance to chemotherapy characterizes these tumors. Consequently, high percentage of local recurrences is observed in chordoma patients [3].

Due to the low incidence of chordomas the molecular pathogenesis of these tumors remains largely unclear. Although, significant progress has been made in that field over the recent years with a few important studies on the role of genomic mutations in chordomas [4–10]. They revealed genetic changes in known tumor-related suppressors and oncogenes such as *PIK3CA*, *PTEN* and *CDKN2A* as well as alterations in tissue-specific genes such as *TBXT* duplications and protein truncating mutations in *LYST* gene [4, 10]. Importantly, the genetic changes were also found in genes encoding proteins involved in epigenetic regulation including recurrent mutations in *PBRM1* and *SETD2* [4, 5, 10].

Contrary to the role of genetic abnormalities little attention was paid to the role of epigenetic changes. Although methods for analyzing DNA methylation profile in humans have been available for years, only very few papers on the genome-wide DNA methylation have been published to date [11–14]. These studies showed changes in chordoma DNA methylation profile in comparison to their normal counterparts [12] and very recently a prognostic relevance of genome-wide DNA methylation pattern was shown [13, 14].

Moreover, some light has also been shed on the transcriptomic definition of chordoma [15–17]. Some researchers were struggling to identify any subgroups within their populations [15], while others identified two gene expression-based clusters [18]. Comparisons of gene expression profiles of tumors with those of the nucleus pulposus (NP, central component of the intervertebral disc, a notochord remnant) samples have revealed differences in expression of brachyury (*T*) [15, 16], *SAMD-5* [16], and other genes associated with development [15]. A recent multi-omic study of chordoma cell lines identified *CA-2* and *THNSL2* as potentially druggable genes in chordoma [17].

The aim of this study was to investigate genome-wide DNA methylation changes and whole-transcriptome expression profile in skull base chordomas. To the best of our knowledge, this is the first research to examine the relationship between the DNA methylation and gene expression profiles of chordoma tumors.

## Materials and methods

### Patients and tissue samples

Thirty-two patients with skull base chordoma were enrolled. They were treated with transnasal and/or transoral endoscopic surgery at the Department of Neurosurgery, Maria Sklodowska-Curie National Research Institute of Oncology, Warsaw, in years 2014–2020. Each tumor sample was split and one part of the tissue was used for routine diagnostic procedures while the second one was snap frozen in liquid nitrogen and stored for molecular analysis. Histopathological diagnosis of a classical chordoma was confirmed according to WHO criteria [19] was confirmed for all the tumor samples. Overall patients’ characteristics are shown in Table 1.

**Table 1:** Characteristics of skull base chordoma patients

Number of patients	n = 32
*Sex*	
Females	15/32 (47%)
Males	17/32 (53%)
*Age [years; median (range)]*	60 (23–76)
Skul base location	
Clivus	21/32 (65.6%)
Clisvus and craniovertebral junction	11/32 (34.4%0
*Surgery type*	
Endoscopic endonasal	31/32 (96.9%)
Craniotomy	1/32 (3.3%)
*Gross resection rate*	
Complete	11/32 (34.4%)
Subtotal	16/32 (50%)
Partial	5/32 (15.6%)
*Recurrence status*	
Newly diagnosed	22/32 (69%)
Recurrent	10/32 (31%)
Tumor size—max. diameter [mm; median (range)]	8,5 (1–17)
*Histological type*	
Classical chordoma	32 (100%)
*Death status*	
No	19/32 (59%)
Yes	13/32 (41%)
Follow up [months—median (range)]	38 (6–97)

Four samples of nucleus pulposus (NP) were obtained from intervertebral disks, collected during discectomy of 4 patients, suffering from degenerative lumbar spine disorder. Nucleus pulposus samples were enzymatically digested for 4 h at 37 °C with 0.2% collagenase type II (Sigma–Aldrich) in a serum-free DMEM, according to a previously validated protocol [20]. The digested tissue/cell suspension was filtered through sterile nylon fabric to remove remaining tissue debris. The cells were subsequently centrifuged at 300× *g* for 5 min and subjected to DNA and RNA isolation.

The study was approved by the local Ethics Committee of Maria Sklodowska-Curie National Research Institute of Oncology in Warsaw, Poland. Each patient provided informed consent for the use of the tissue samples for scientific purposes.

### Nucleic acid isolation

Genomic DNA and total RNA from tissue samples were isolated using AllPrep DNA/RNA/miRNA Universal Kit (Qiagen). The procedure included tissue homogenizing with rotor stator homogenizer Omni Tissue Master (Omni International). The concentration of nucleotides was measured both spectrophotometrically using NanoDrop 2000 (Thermo Scientific) and with fluorescence-based method using QuantiFluor Dye kit (Promega) and Quantus (Promega) instrument. Isolated total RNA was stored at − 80 °C, whereas genomic DNA was − 20 °C.

### Genome-wide DNA methylation profiling

DNA from 32 skull base chordomas and 4 NP samples were bisulfite converted with EZ-96 DNA Methylation kit (Zymo Research) and used for genome-wide DNA methylation profiling with Methylation EPIC (Illumina) BeadChip microarrays. Recommended protocol for Infinium MethylationEPIC Kit was used (Infinium HD Methylation Assay Reference Guide, Illumina). Laboratory procedures were performed by the Eurofins Genomics service provider.

### Whole transcriptome sequencing

Whole-transcriptome expression profile based on RNA sequencing (RNA-seq) was determined for 32 skull base chordoma and 4 NP samples. One μg of total RNA from each tissue sample was used for library preparation with NEBNext Ultra II Directional RNA Library Prep Kit for Illumina (New England BioLabs). NEBNext rRNA Depletion Kit was applied for ribosomal depletion. The quality of libraries was assessed using the Agilent Bioanalyzer 2100 system (Agilent Technologies, CA, USA). Libraries were then sequenced on an Illumina NovaSeq 6000 platform, and 150-bp paired-end reads were generated. A minimum of 30 M read pairs per sample were generated. Sequencing was performed by the Eurofins Genomics service provider.

### Immunohistochemical staining

Immunohistochemical staining (IHC) was performed on 4-μm FFPE tissue sections using Envision Detection System (DAKO/Agilent, Glostrup,), according to manufacturer’s protocol. Tissue sections were deparaffinized with xylene and rehydrated in a series of decreasing concentration ethanol solutions. Heat-induced epitope retrieval was performed in a 96 °C water bath, for 30 min in Target Retrieval Solution pH 9 (DAKO). Tissue samples were incubated with the primary antibodies against CD3 (clone F7.2.38; dilution 1:50; DAKO/Agilent) CD8 (clone D8A8Y; dilution 1:200; Cell Signaling Technology) and CD4 (clone; dilution 1:500; Cell Signaling Technology) for 1 h in RT. Color reaction product was developed using 3,3′-diaminobenzidine tetrahydrochloride as a substrate and nuclear counterstaining was obtained with hematoxylin.

### Data analysis

Data were analyzed, utilizing R statistical programming language (version 4.2.2). Code is freely available in a GitHub repository (https://github.com/SBaluszek/chordoma_RNA_met).

DNA methylation was analyzed using minfi [21]—all samples passed quality control. Methylation probes were filtered (SNP, probes that have failed in at least 25% of samples) and normalized, utilizing *funnorm* function. Top one percent probes with β-value standard deviation above 0.1 were utilized for gaussian mixture modelling-based clustering [22], for cross-validation hierarchical clustering (euclidean distance and ward.D method, base R package) was utilized. Subsequently, probes differentially methylated between clusters, nucleus pulposus, and chordoma were identified using minfi [21]; p-values lower than 9e−8 were considered significant [23]. The M-values distributions were tested in a linear model and related β-values were visualized with violin plots. Subsequently, differentially methylated regions (DMRs) were identified with comb-p [24].

Reads abundance on gene and transcripts levels were quantified using kallisto [25] on GRCh37 patch 13 cDNA sequences, downloaded from Ensembl genome database (http://grch37.ensembl.org/Homo_sapiens/Info/Index). Differential gene expression and gene expression normalization was performed, utilizing DESeq2 [26]. Clustering of genes most variable, selected by squared coefficient of variation [27], was performed with gaussian mixture modelling-based clustering [22], for cross-validation hierarchical clustering (euclidean distance and ward.D method) was used. Interaction between methylation and gene expression data was analyzed by means of Kendall correlation of averaged M-values within 1500 bps transcription start site (TSS) and scaled gene expression. Methylation-controlled genes were thusly identified. A similar approach was utilized for DMRs (with mean M-values as proxy for DMR methylation level).

Subsequently, differences in gene expression fold-change were analyzed utilizing Fast Gene Set Enrichment Analysis [28] with Gene Ontology and Reactome terms, downloaded from MSigDB [29, 30]. Furthermore, weighted correlation network analysis (WGCNA) [31] was utilized to obtain modules of genes, whose association with each methylation cluster was tested with U-Mann–Whitney test. Subsequently, selected modules were intersected with STRING a protein–protein interaction database [32]. Importance of genes in the network was inferred using WGCNA connectivity measure and authority centrality [33], provided by tidygraph package [https://tidygraph.data-imaginist.com].

The tumor microenvironment was deconvoluted from DNA methylation and RNA sequencing—utilizing MethylResolver [34] and immunedeconv [https://github.com/omnideconv/immunedeconv], respectively. MCPcounter [35] and ESTIMATE [36] were implemented, using immunedeconv. The latter was also used in order to compare chordoma clusters with the common human cancer types. Subsequently, non-parametric statistical tests were utilized, where appropriate.

DNA copy number changes were inferred in two-fold manner—conumee was run on the methylation data [37] and gsealm [38] with data for chromosome bands from MsigDB [29, 30]. Non-parametric statistical tests were utilized, where appropriate and Cox proportional-hazard model was utilized to test for the survival outcomes.

Results were validated by comparison with a dataset available from Gene Omnibus GEO (GSE205331) [14]. For reanalysis purposes, data were filtered and normalized with *funnorm* as described previously. Authors of GSE205331 have utilized top 10 000 most variable probes to construct their clusters and those were combined with 3648 probes from our study. However, due to differences between EPIC and 450 K methylation microarrays, only 10,010 probes were available in both datasets and those were utilized downstream. Hierarchical clustering was utilized for unsupervised analysis*.* For global methylation 325,137 probes common in both assays with β-value standard deviation above 0.1 were utilized. U-Mann–Whitney-Wilcoxon was utilized for comparisons of probes of known significance in chordomas and MethResolver estimates, computed for the combined dataset. Uniform Manifold Association Projection was utilized for samples visualization in Additional File 5: Figure S5.

All visualizations were performed with ggplot2 R library [https://ggplot2.tidyverse.org].

## Results

### Chordoma genome-wide DNA methylation profile

**Fig. 1:**
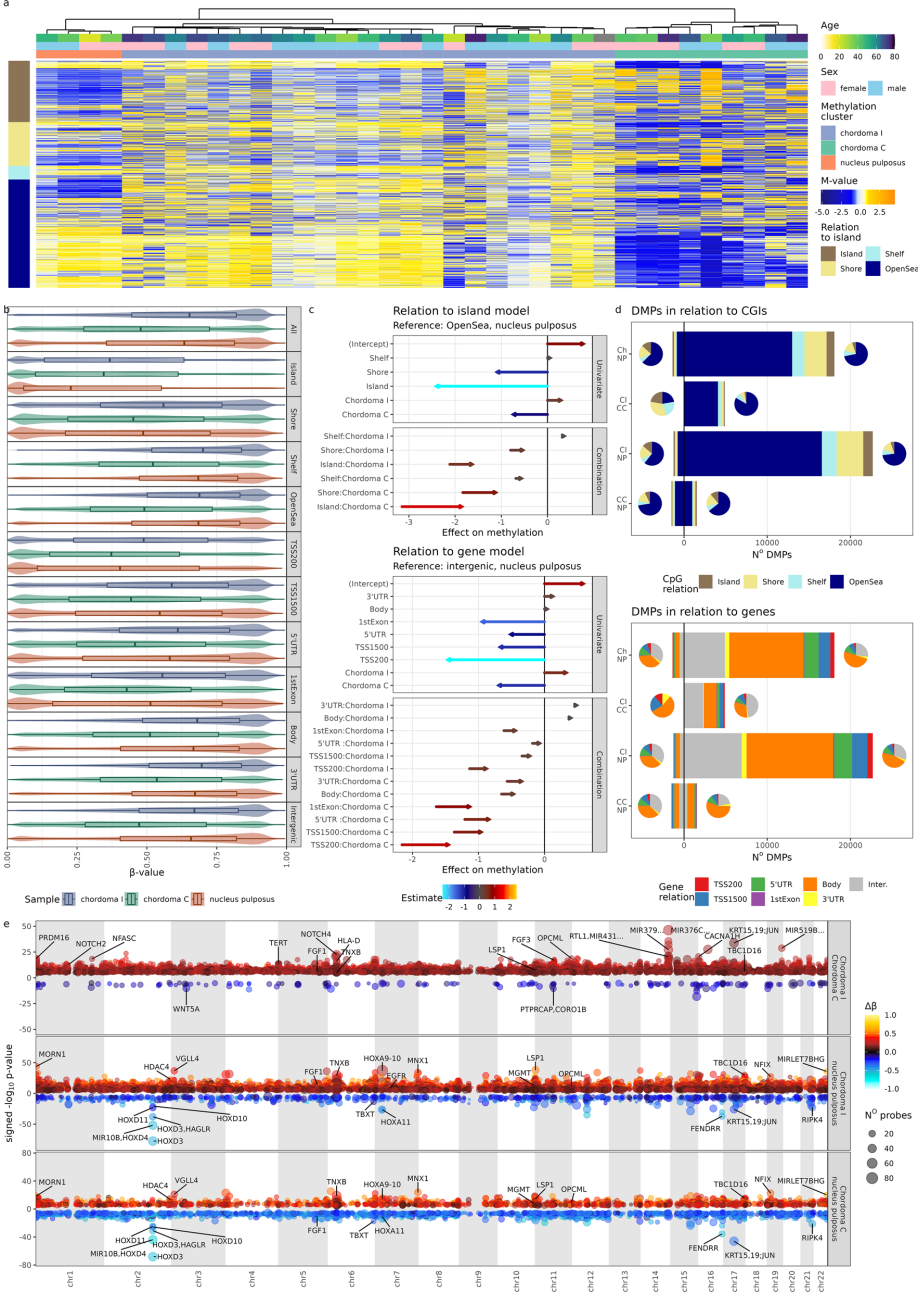
Results from EPIC DNA methylation arrays **a** Heatmap of scaled methylation M-values of 3648 most variable probes, split in rows, according to CpG relation to CGI and clustering of samples. **b** Beta values of 364,784 probes with standard deviation of β-values above 0.1, split according to CpG relation to CGI and gene. **c** Quantification of differences in overall methylation levels using a linear model of M-values. **d** Distribution of differentially methylated probes classified according to their position regarding CGI and genes. **e** Differentially methylated regions, depicted on Manhattan plots (genomic position on x-axis), gene names for most significant and biologically interesting DMRs are captioned

Following quality control, a set of 834,918 array probes (excluding SNP regions and probes with probe detection *p* value above 0.05) were analyzed in all 32 samples of skull base chordoma and 4 NP samples. Subsequently, top 3648 most variably methylated probes (top one percent of variable probes from set of probes with standard deviation of β-values above 0.1) were clustered with two independent clustering methods i.e. hierarchical clustering and gaussian modelling-based clustering. Both methods revealed presence of three separate clusters: one containing 4 NP samples and two clusters composed of chordoma samples. The larger one (23 samples) was called chordoma I and the smaller one (9 samples) chordoma C [13] (Fig. 1a). Interestingly, results of hierarchical clustering indicate that chordoma I samples were more similar to NP than to chordoma C samples.

Genome-wide DNA methylation was notably lower in chordoma C than in NP. However, this was most prominent in probes located in the *Open Sea* regions as depicted by methylation pattern of top variably methylated probes (Fig. 1a). This initial observation was further investigated in a broader set of variable probes (364,784 probes with standard deviation of β-values above 0.1; Fig. 1b, c). This confirmed that general hypomethylation in cluster C chordomas was most pronounced in the *Open Sea* regions. Hypomethylation was less apparent in CpGs closer to CpG islands (CGIs) (including CGI *shelves* and *shores*) while the probes in CGIs were hypermethylated in comparison to NP. In turn, chordoma I cluster was globally only slightly hypermethylated; however, this effect is more pronounced in CGIs and *Shore* regions (Fig. 1b). When relation of DNA methylation to genes was considered, a more nuanced image emerged—chordoma I samples clearly displayed hypermethylation in promoters, whereas chordoma C samples varying levels of hypomethylation. In both gene bodies and intergenic regions, chordomas from C cluster were clearly hypomethylated (Fig. 1b).

In order to quantify these observations, a linear model on M-values was built. Its results, depicted in Fig. 1c, warrant explanation: e.g. methylation levels were compared with nucleus pulposus using Open Sea samples/probes as reference. Based only on that, hypomethylation in chordoma C and CGIs would be expected. However, as described earlier, this was not the case, and therefore the effect estimation for combination of chordoma C and Island was strongly positive. As chordoma I samples were generally more methylated, this combination estimate had lower value. Similar but slightly weaker effect was seen in probes relation to genes—probes located on 5’ end (5’UTR, TSS1500, TSS200 and 1st exon) were globally hypomethylated, as were chordoma C samples. However, this combination effect was positive, translating into methylation levels comparable to NP in these 5’ end probes. This model provides formal evidence to the previously made claims and demonstrates that similar hypermethylation of promoters, relative to the intergenic regions, occurred in chordoma C samples, however, the effect size was smaller and consequently globally hypomethylated pattern dominates at promoters in this cluster.

Subsequently, differentially methylated probes (DMPs) between two chordoma clusters, as well as between chordomas and NP were identified (listed in Additional File 7: Table S1, summarized in Table 2). Accordingly with results showing higher global DNA methylation in chordoma I than chordoma C subtype, vast majority (9 to 4892) of DMPs identified in comparison of two subtypes of tumors were the probes hypermethylated in I chordomas. More detailed designation of DMPs, according to their location in relation to the CGIs and genes is depicted in in Fig. 1d—even though chordoma C hypermethylation was most pronounced in the promoters and islands, majority of DMPs was found in the Open Sea and gene bodies. This probably can be explained by the general distribution of probes in the EPIC array and discrepancy in cluster size (as cluster I is bigger, more probes, differing in chordoma versus NP comparison, pass the significance threshold). Furthermore, a general pattern of global hypermethylation of chordoma I is reflected by the number of hypermethylated probes in chordoma C in comparison with nucleus pulposus.

**Table 2:** The numbers of differentially methylate probes and regions as wells as differentially expressed genes for each chordoma of nucleus pulposus sample group

First group	Second group	Differentially Methylated Probes	Differentially Methylated Regions	Differentially Expressed Genes	WGCNA modules
Chordoma I	Chordoma C	4892	13622	1128	4
Chordoma C	Chordoma I	9	180	170	0
Chordoma I	Nucleus pulposus	22676	15705	5078	12
Nucleus pulposus	Chordoma I	1261	1208	4052	11
Chordoma C	Nucleus pulposus	1532	2825	3426	8
Nucleus pulposus	Chordoma C	1502	3245	3426	9
All chordomas	Nucleus pulposus	18064	11670	5293	13
Nucleus pulposus	All chordomas	1383	1083	4252	13

The DMPs were aggregated into differentially methylated regions (Fig. 1e and Additional File 6, which allows reader to interactively explore all DMRs). There were far more hypermethylated DMRs in chordoma I than chordoma C samples both in direct comparison of two subtypes of the tumors and in comparison of each subtype with NP. DMRs with most significant difference between groups of samples and DMRs located in the neighborhood of known cancer-related genes were marked in Fig. 1e. Comparison of two chordoma subtypes showed that most significant DMRs are located on chromosome 14 and 19 regions containing microRNA clusters as well as on chromosome 17 region coding for *KRT15*, *KRT19* and *JUN*. These regions are hypermethylated in chordoma I, when compared to chordoma C.

DMR identified in comparison of chordoma and NP were located in genes with known role in tumor biology, including *TERT, BLM, CDH11, CDH4, DLC1, OPCML, HIF1A, YWHAQ, MGMT, TP63, MTOR, MUPCDH, RIPK4*, *EGFR* or *TBC1D16.* Most of these DMRs are significantly more hypermethylated in chordoma I (Fig. 1e, Additional File 6). Chordoma-specific DMRs were also found in location of homeobox domain genes (e.g. DMRs in *HOXA4, HOXA5*, *HOXD3*, *HOXD4 MNX1*, and *NFIX*) especially on chromosome 2 at *HOXD* cluster. Moreover, DMR was also identified in *TBXT* (T) that encodes brachyury—a notochord-specific transcription factor that plays developmental role. DMR *in TBXT* was hypomethylated in both chordoma clusters as compared to NP.

### Gene expression in chordoma DNA methylation clusters

Gene expression profile was determined in each chordoma and NP sample based on RNA-seq data. An average 41,315,537 reads per sample were obtained with average 79.65% reads pseudoaligned to UCSC hg19 cDNA transcriptome. The sequencing reads were quantified on 39,293 human transcripts. Of those, 23,013 passed the filtering criteria (at least 5 reads in at least 10 samples) for normalization and differential expression analysis. Number of differentially expressed genes for each condition is presented in Table 2.

**Fig. 2:**
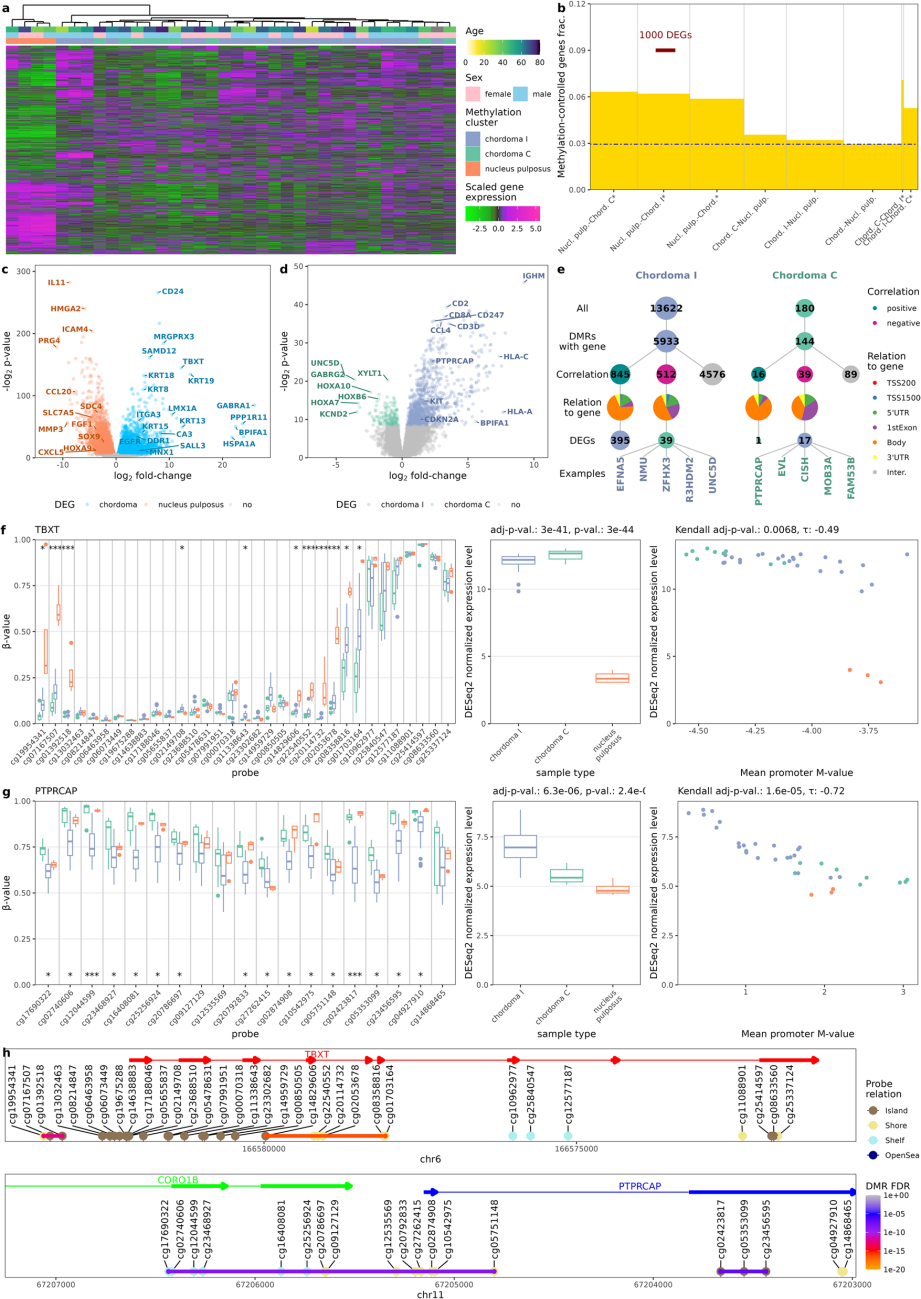
Analysis of genes expression and its relation to the DNA methylation profile. (**a**) Heatmap of 4275 top most variably expressed genes and their clustering showing lack of clear overlap between methylation and expression-based chordoma clusters (**b**) Fraction of genes differentially expressed between chordoma subtypes as well as chordomas and nucleus pulposus that are under DNA methylation control (as defined by significant correlation of mean promoter methylation with gene expression), the blue dot-and-dash line indicates the level of methylation control of the genome in general (**c**) Volcano plot of differentially expressed genes identified in chordoma—nucleus pulposus comparison (**d**) Volcano plot of differentially expressed genes found in chordoma I—chordoma C comparison (**e**) DMR relation to DEGs in chordoma I—chordoma C comparison, height of the bars represents number of genes/DMRs (**f**) Role of DNA methylation in TBXT (brachyury) gene. Difference in the methylation levels of CpGs at *TBXT* locus with DMPs labeled with *(for adj.p < 0.05), **(for adj.p < 0.001) or ***(for p-value <9*10^−8^) (left panel), difference in *TBXT* expression of (middle panel) and correlation between *TBXT* promoter methylation and expression levels (right panel); p-values are shown for chordoma-nucleus pulposus comparison (**g**) Difference in the methylation levels of CpGs at *PTPRCAP* locus with DMPs labeled with *(for adj.p < 0.05), **(for adj.p < 0.001) or ***(for p-value < 9*10^−8^) (left panel), difference in *PTPRCAP* expression of (middle panel) and correlation between *PTPRCAP* promoter methylation and expression levels (right panel) (**h**) Position of *TBXT* (brachyury) and PTPRCAP genes on their chromosomes, along with EPIC methylation probes position and DMR

Based on squared coefficient of variation model [27], 4275 most variable genes were selected and hierarchical clustering was performed. One major chordoma expression cluster, including 30 samples, was observed with the remaining 3 samples and NP in two remaining separate clusters (Fig. 2a). No apparent relation to the methylation cluster was observed. Nonetheless, to investigate further, entanglement between DNA methylation- and gene expression-bases clusters was measured. The resulting of entanglement 0.22 indicates that, despite general differences between methylation and expression-based clustering, clustering structures are not entirely dissimilar, pointing to more discrete correlations at few-sample level (Additional File 1: Figure S1). Furthermore, fraction of genes, remaining under methylation control was determined (Fig. 2b). Gene was classified as methylation-controlled, when correlation of mean promoter methylation and gene expression was significant and negative, as it is commonly done [39]. In general, 2.9% of genes fulfilled these criteria. However, this fraction was significantly higher in genes differentially expressed in chordoma (Fig. 2b). This indicates that DNA methylation pattern in chordomas influences gene expression on a global scale.

Volcano plots of differentially expressed genes between chordoma and nucleus pulposus samples are shown in Fig. 2c and between chordoma I and chordoma C samples in Fig. 2d. Genes marked on the volcano plot were either implicated earlier in chordoma biology (e.g. keratins—*KRT8*, *KRT18*, *KRT19*, *TBXT*, *LMX1A*, and *EGFR*) or identified by outstanding p-value (e.g. *IL11*, *CD24*), high absolute fold-change (e.g. *GABRA1*) or DMR result (e.g. *MNX1*). Genes demonstrated on this plot tend to belong to three general categories: immune response-related (e.g. *IL11*, *ICAM4*, *CXCL5*), involved in notochord development (e.g. *TBXT*, *MNX1*, *HOXA9*, *SOX9*), and epithelial or connective tissue-specific (keratins, *SDC4*, *PRG4*). In the case of comparing clusters with each other, overlap with existing chordoma cluster markers was less striking—only *KIT* and *CDKN2A* were overexpressed in chordoma I. Other genes, related to this cluster, were skewed towards immune infiltration (HLA proteins, leukocyte markers, cytokines) and high *KIT* expression can be also considered to be related to immune cell signaling. *CDKN2A* loss was previously described in chordoma [40] and evidence of this phenomenon was also seen in further analysis in chordoma C cluster (see section DNA copy number changes in two epigenetic subtypes of chordomas and Fig. 5). Furthermore, higher expression levels of homeobox-containing genes were observed in chordoma C.

The relation between DMRs and DEGs was examined and is presented in Fig. 2e, which also illustrates our investigative process. Out of 13,662 and 180 hypermethylated DMRs, 5933 and 144 were located in gene-annotated region in chordoma I and chordoma C respectively. In both cases, most of them did not correlate with gene expression. Of those that did, positive correlation was associated with DMRs located in the gene body—this is not surprising, given that gene body methylation is associated with gene transcription [41]. Negative correlation of methylation and gene expression was observed in more promoter-adjacent regions and of 512 and 39 genes negatively correlating with a DMR 39 and 17 were DEGs in chordoma I and chordoma C. Five such genes from each group, with highest τ correlation coefficient are shown on Fig. 2e and PTPRCAP was investigated further (Fig. 2g, h). Detail results of this DMR-gene expression correlation analysis are available in Additional File 7: Table S7.

Similar correlation analysis was performed for DEGs and DMRs that were found in comparison of chordoma and NP samples. It showed that expression of 1033 DEGs is correlated corelated with methylation level of total 1543 DMRs. Mainly negative methylation/expression correlation was found as it was observed in case of 755 of expression-related DMRs.

For genes with at least moderate correlation (correlation coefficient > 0.3) between DNA methylation and expression level we ran overrepresentation analysis with Gene Ontology and Reactome databases to investigate if there are specific pathway enrichment of the methylation-controlled DEGs. When analyzing methylation-correlated genes that are differentially expressed in chordoma I and C subtypes we found the enrichment in particular terms, related basically to immune inflammation and signaling by Rho GTPases. Overrepresentation analysis of DEGs with a corresponding DMR found in comparison of chordoma versus NP showed the enrichment of terms related mainly to extracellular structure organization, cellular junction, epithelial/mesenchymal transition. The significantly enriched terms are listed in Additional File 7: Table S7.

Among the aberrantly methylated and expressed genes, special focus was placed to two genes—*TBXT* (brachyury, with official symbol T) and *PTPRCAP*. Brachyury is already recognized as a chordoma diagnostic marker. According to our data, its expression is correlated with methylation in the promoter (Fig. 2f). CGIs best differentiating chordoma from nucleus pulposus flank the *TBXT* gene promoter and two DMRs were identified up- and downstream to the promoter (Fig. 2h, which serves as a genomic map of probes and DMRs). On the other hand, *PTPRCAP* is only one of many immune-related genes, characteristic of chordoma I. It was selected, due to its high promoter methylation-expression correlation (Fig. 2g). All probes, overlapping with the gene were identified as DMR (Fig. 2h).

### Gene set enrichment and weighted correlation network analyses

**Fig. 3:**
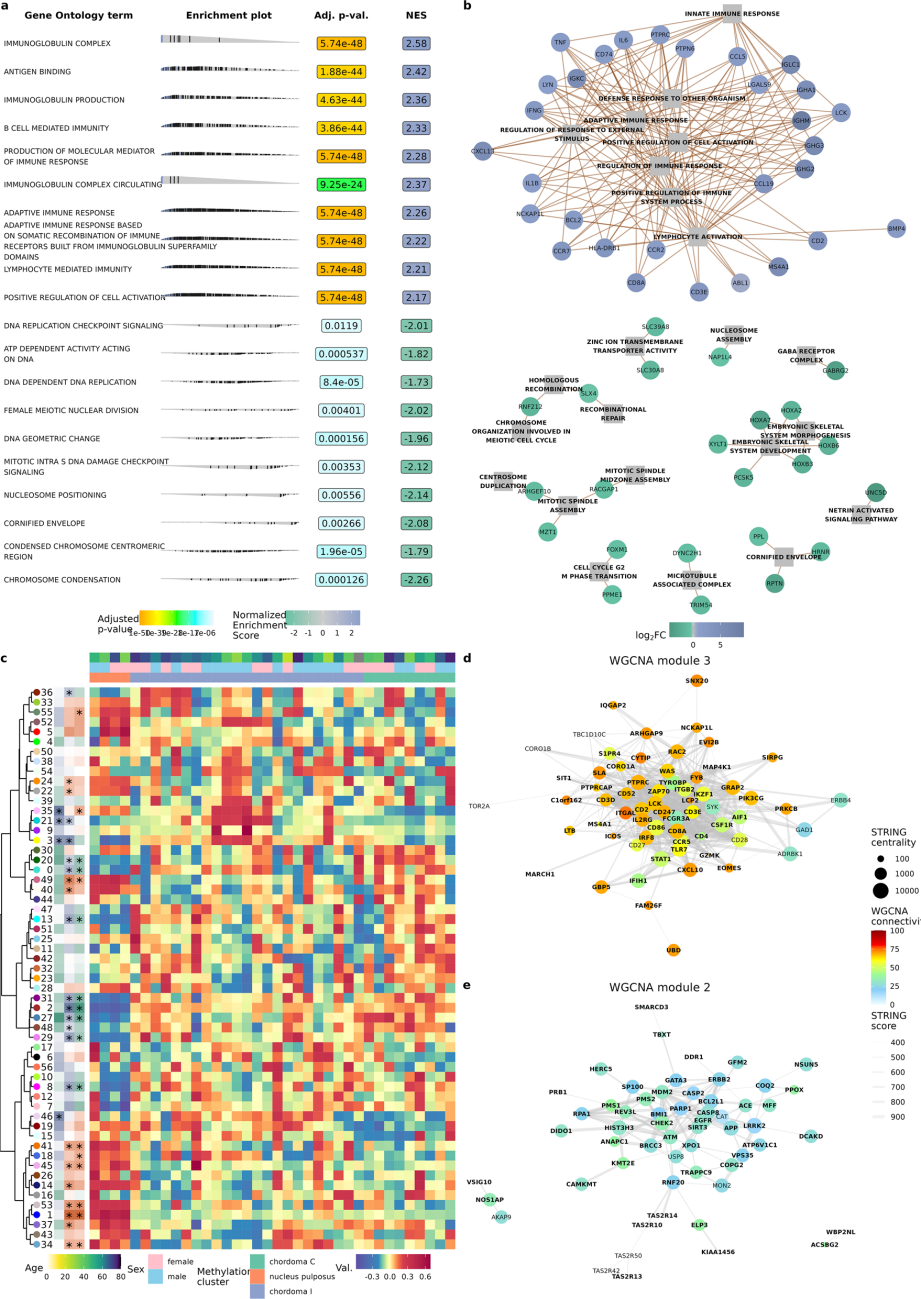
Functional analysis of the RNA sequencing results (**a**) Top 20 terms of gene set enrichment analysis on gene ontology terms (chordoma I–chordoma C comparison) (**b**) Differentially expressed genes and terms from gene set enrichment analysis. Due to number of terms and genes top hits, basing on p-value, were selected (**c**) Weighted gene co-expression network analysis, presented on heatmap; samples are shown in columns, WGCNA modules in rows, significance of tests is shown in the panel on the left (**d**) WGCNA module 3 (with significantly higher score for chordoma I samples) intersected with STRING database; genes to plot were selected based on top connectivity (importance for the module) and centrality (best connected within module); gene names in bold were also differentially expressed in chordoma I, compared with chordoma C (**e**) Analogous for module 2, higher in chordoma versus nucleus pulposus, DEGs for chordoma—NP comparison are shown in bold

To characterize functional differences between the two chordoma clusters and NP, gene set enrichment analysis (GSEA) with terms from Gene Ontology (GO, Fig. 3a, Additional File 7: Table S4) and Reactome (Additional File 7: Table S4) databases was performed. The results displayed remarkable difference between clusters I and C—genes overexpressed in chordoma I were enriched in terms associated with immune response, with terms associated with adaptive immune response on the very top of the list. Genes sets, characterizing cluster C, were more heterogenous and included terms related to cell cycle and proliferation with keratinization process, and developmental pathways (Fig. 3a). The top terms from gene set enrichment analysis of DEGs is presented in Fig. 3b. Probably stemming from the characteristic of chordoma I cluster (which outnumbered chordoma C cluster 23 to 9), the main difference between chordoma and nucleus pulposus were associated with immune system terms. Terms associated with cartilage and connective tissue differentiation characterized NP samples, possibly pointing to deficiency of those processes in chordoma.

Subsequently, weighted correlation network analysis (WGCNA) was undertaken in order to disentangle gene–gene correlations and operate on fewer, easier to understand modules. Fifty-six such modules were identified (Fig. 3c, Additional File 2: Figure S2). Modules were clustered and differences between chordoma methylation clusters and between each of them and NP were tested (see Table 1 for summary). A cluster of modules 2, 27, 29, 31, and 48 had consistently higher eigenvalues in chordomas while cluster of modules 3, 21, and 35 was associated with chordoma I. Modules 2 and 3 had lowest p-value and log2 fold-change in respective comparisons. In order to validate this finding, clusters were overlapped with protein–protein interactions in STRING database. Notably, *TBXT* was found in module 2 and *PTPRCAP* in module 3. Both modules were significantly enriched in protein–protein interactions (*p* < 1e−16). Genes, treated as nodes in network were assigned centrality measures (centrality measures indicate how important is the gene for the network; see methods for details) and WGCNA connectivity scores, which answer a question of how much intra- versus extramodular correlations each gene has. Due to a large number of genes in modules (2690 in module 2 and 2109 in module 3) only genes with high centrality or connectivity scores are shown in Fig. 2d and e. Genes central in module 3 were immune response-related (e.g. *CD247*, *CD3E*, *CD8A*, *IL2RG*, and *ITGAL*). Genes central for module 2 (and thus probably important for functioning of chordomas in both clusters) were either responsible for cell division and DNA repair process (e.g. *ATM*, *CHEK*, *BMI1*, *MDM2*) or parts of inhibitors of apoptosis cascade (e.g. *CASP2*, *CASP8*, *SIRT2*).

### Immune infiltration in chordomas

**Fig. 4:**
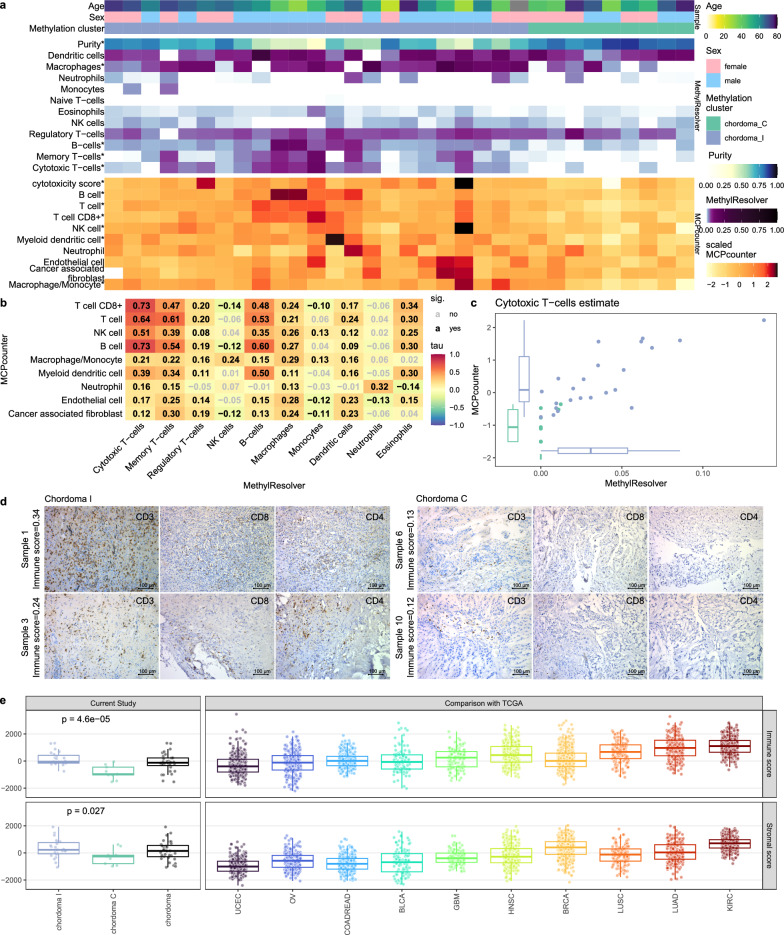
Cell type deconvolution of RNA-seq and DNA methylation data (**a**) heatmap presenting scores from two independent methods—MethylResolver (utilizing EPIC DNA methylation data, upper panel) and MCPcounter (utilizing RNA-seq data, lower panel) both point to higher immune infiltration in chordoma I cluster, significant differences in U-Mann–Whitney test are marked with * (**b**) Correlation matrix of immune signatures from both methods (Kendall correlation); coefficients are shown in the middle of each cell, significant ones are black (**c**) Plot presenting signature correlation for cytotoxic T-lymphocytes from both methods, correlation coefficient: 0.73, adjusted *p* value: 2.8e−142, cluster differences adjusted *p* values: 0.0012 and 0.0003 for MethylResolver and MCPcounter, respectively (**d**) Representative examples of immunohistochemical staining of selected chordoma C and chordoma I samples with antibodies against CD3, CD4 and CD8. (**e**) Comparison of chordomas (including chordoma subtypes classified according to DNA methylation profile) with existing signatures for variable human cancer types from ESTIMATE method (signatures for immune and stromal components of the tumor)

Since GSEA, following differential expression analysis, clearly indicated difference in the immune response between the chordoma clusters, the content of immune cells in chordoma samples was imputed. Deconvolution methods, based on genome-wide DNA methylation data and gene expression profiles were used for this purpose independently. The results of both approaches consistently indicated notable, higher content of immune infiltrating cells in chordomas I than in chordomas C. Chordoma I samples had lower tumor purity in MethylResolver deconvolution (median 0.65 vs median 0.82, *p* = 3.4e−5, Fig. 4a). Specifically, this was reflective of a higher estimated infiltration of cytotoxic T-cells (*p* = 1.4e−4, *p* = 3.0e−4), B-cells (*p* = 6.4e−4, 4.8e−6) and macrophages (*p* = 0.001, *p* = 0.006) in both MethylResolver and MCPcounter approaches (Fig. 4a). A relatively high inter-method scores correlation was observed, i.e. inter-method correlation of abovementioned populations signatures were high (Kendall Τ 0.73, 0.60, and 0.29 respectively; see Fig. 4b). The results, obtained for cytotoxic T-cells in both methods are shown on Fig. 4c.

The difference of immune status of two chordoma clusters was verified by microscopic analysis. Ten samples, including 5 chordoma I and 5 chordoma C tumors, with various immune scores, estimated with deconvolution method, were subjected to IHC staining against CD3, CD4 and CD8 (markers of T cell populations). This showed a higher content of immune cells in chordoma I samples, with higher immune score in comparison to chordoma C tumors and a chordoma I sample with low immune score. Representative staining results are shown in Fig. 4d, while staining results of all the samples are shown in Additional File 4: Figure S4. Chordoma microenvironment was further analyzed in the wider context of other neoplasms. Data from The Cancer Genome Atlas, deconvoluted with ESTIMATE method, were downloaded and scores generated by ESTIMATE method on our expression data were compared to them. Both stromal and immune scores were significantly higher in chordoma I than in chordoma C (Mann–Whitney p: 6e-5 and 0.022, respectively). Comparison with other cancer types revealed amounts immune score in chordomas I comparable to glioblastoma, bladder and colorectal cancer, while chordomas C have median immune score remarkably lower than other human cancers, as visualized in Fig. 4e.

### DNA copy number changes in two epigenetic subtypes of chordomas

**Fig. 5:**
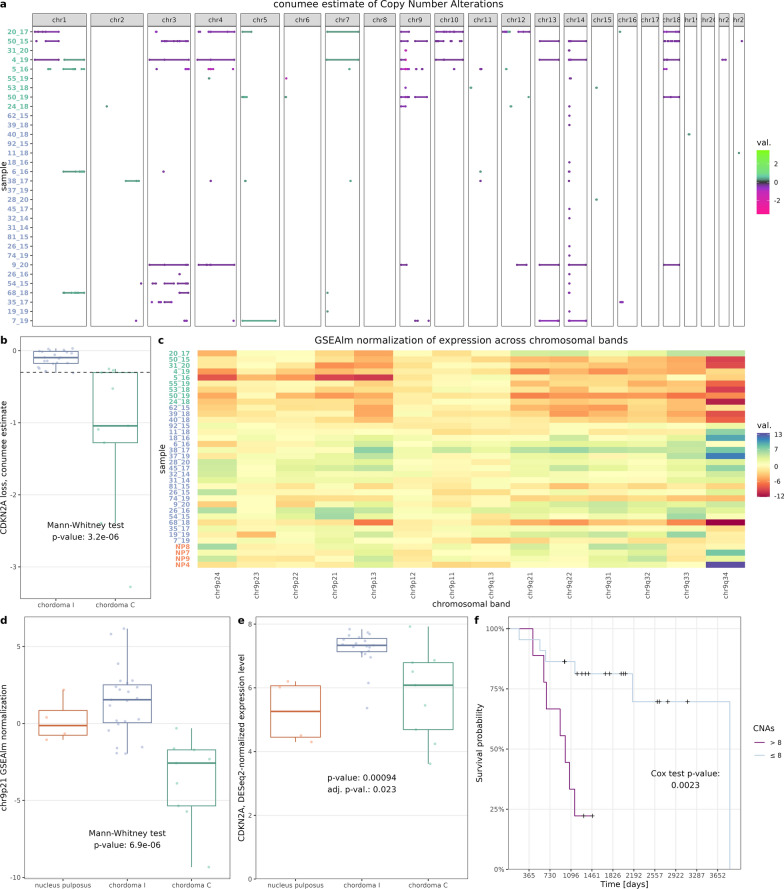
Estimations of copy number alterations and their biological and prognostic relevance (**a**) Results of copy number variation (CNV) imputation from EPIC DNA methylation array performed by conumee method. Genomic segments with absolute scores above 0.3 are shown. Sample label colors correspond to methylation clusters (**b**) Box plot for conumee score of *CDKN2A* gene locus; 0.3 cut-off marked with dashed line (**c**) Estimation of gene expression across selected regions of chromosome 9 (according to chromosomal bands), calculated by GSEAlm package; label colors correspond to methylation clusters (**d**) Box plot of GSEAlm score for chr9p21 band (containing *CDKN2A*/*B* genes*)* that has lowest p-value as the result of comparing two chordoma subtypes in terms of the expression across the whole genome. (**e**) *CDKN2A* expression across methylation clusters and normal nucleus pulposus (**f**) Kaplan–Meier plot for number of CNVs (0.3 cut-off in conumee estimate); Cox proportional hazard model was used for testing, cut-off of 8 CNVs was picked by maxstat package for visualization purposes

EPIC DNA methylation array allows the evaluation of large, unbalanced, structural chromosomal genomic amplifications and deletions (Copy Number Alterations, CNAs). Copy number analysis was applied to chordoma samples and with a cut-off of 0.3 for absolute copy number change; 261 copy number events were identified in all samples (Fig. 5a). The mean number of segments was 7.19 with quantile distribution of [0, 1, 2.5, 11.25, 34]. Moreover, CNAs were more common in chordoma C cluster as compared to chordoma I (median 14 vs 1, *p* = 0.02).

The main difference in CNAs between two chordoma clusters were chromosomic losses in chromosome 9 region, containing *CDKN2A/B*, affecting chordoma C cluster. This loss was found in 9 of 10 tumors from chordoma C cluster while it was observed only in 1 out of 23 tumors of cluster I, with CNA score cut-off at 0.3 (exact Fisher test *p* = 7.95e−5). This difference is also significant when comparing *conumee* scores for this region (Fig. 5b). In order to verify the functional consequences of chromosome 9 deletions, expression levels of genes across chromosomal bands was examined. Expression of genes within 9p21 band (containing *CDKN2A*) was the most significantly different and lower in chordoma C (Fig. 5c and 5d, Additional File 3: Figure S3). As mentioned earlier, *CDKN2A* was overexpressed in chordoma I cluster (Fig. 5e).

### Prognostic factors in chordomas

Various features, including methylation cluster, immune infiltration scores, WGCNA modules, and *CDKN2A* loss were tested for effect on overall survival. However, only the total number of CNAs showed a significant association with survival—patients with more variations had a higher risk of death (Fig. 5f). This effect was seen in both uni- and multivariate analysis (Table 3).

**Table 3:** Survival effect of selected clinical and molecular features in a uni- and multivariate Cox hazard model

Variable	Trait	Multivariate			Univariate		
		Coefficient	Z	*p* value	Coefficient	Z	*p* value
CNVs		0.16	2.92	0.003	0.08657	3.05	0.002
Immune infiltrate		6.70	1.60	0.11	− 2.32	− 1.06	0.29
Sex	Male	− 1.12	− 1.45	0.15	0.05	1.84	0.07
Age		0.04	1.40	0.16	− 0.45	− 0.77	0.44
Cluster	Chordoma I	0.60	0.61	0.54	− 0.32	− 0.52	0.60
		Likelihood ratio test = 15.56 on 5 degrees of freedom, *p* = 0.008 events: 13 of 31 observations					

### Validation of the findings

**Fig. 6:**
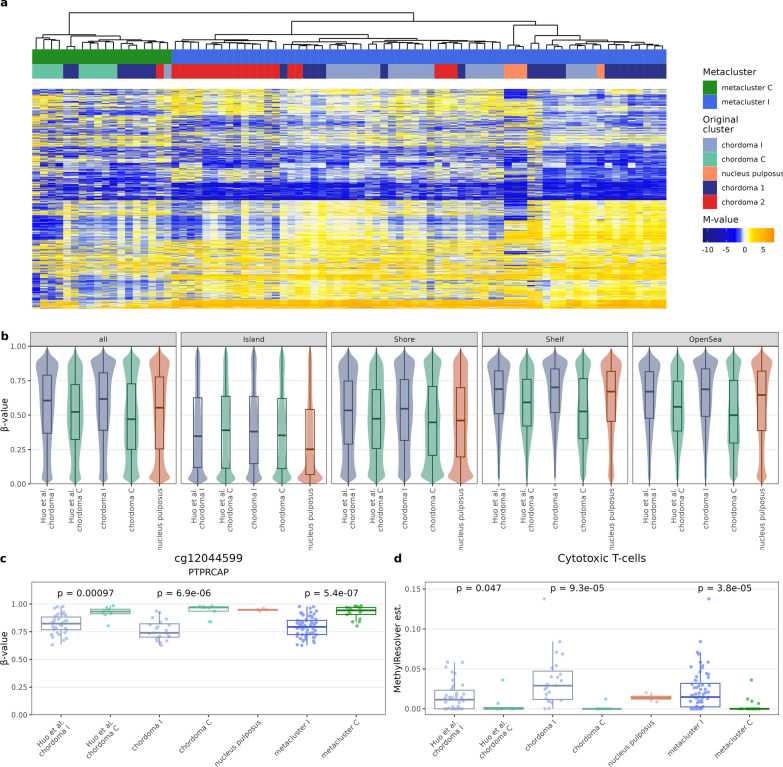
Validation of the study findings in an independent data set (GSE205331) [14] (**a**) Clustering the samples with a set of 10 010 array probes (variable probes from both experiments, occurring in both EPIC and HM450K arrays) with heatmap of scaled methylation M-values (**b**) Beta-values of probes with standard deviation of β-values above 0.1, split according to CpG relation to CGI (**c**) Difference in the methylation levels of cg12044599 CpG in *PTPRCAP* locus in chordoma C and I subtypes in samples from current study, validation data set and in metaclusters (**d**) Difference in the content of CD8^+^ lymphocytes in chordoma C and I subtypes from current of study group, validation data set and in metaclusters

For cross-validation of our findings classification system into chordoma C and chordoma I subtype was applied to independent dataset from DNA methylation profiling with HM450K arrays in 48 chordomas (GSE205331)[14]. A set of 10 010 array probes (variable probes from both experiments, occurring in both EPIC and HM450K arrays) were utilized for construction of consensus M-value matrix for unsupervised analysis. Pooled samples from both datasets were clustered in the same manner as in the investigation phase of the study and a clear distinction into two clusters was observed (Fig. 6a). A less numerous cluster (metacluster C), contained 18 samples, including all 9 our chordoma C, 7 chordoma 1 samples, 1 chordoma I sample, and 1 chordoma 2 sample. The larger metacluster C contained remaining 60 samples (22 chordoma I, 20 chordoma 1, 18 chordoma 2, and 4 nucleus pulposus samples).

The differences between chordoma C and chordoma I samples from validation set are concordant with those observed in our samples. In order to validate the global methylation effects, a consensus β-values matrix of common probes with standard deviation of β-values above 0.1 was constructed. Again, global hypomethylation of chordoma C was most pronounced in the Open Sea region, less pronounced in the CGIs with gradient of this effect in Shelves and Shores (Fig. 6b). Similar concordance was observed with regard to methylation probe relation to gene (Additional File 5: Figure S5a). Moreover, hypomethylation of cg12044599 probe upstream of *PTPRCAP* promoter in chordoma I was observed in both validation set and metacluster I (Fig. 6c). The cg01392518 probe upstream of *TBXT* promoter had also virtually no methylation in chordomas from validation set (Additional File 5: Figure S5c). In *MethylResolver* analysis, CD8^+^ lymphocytes were also more abundant in chordoma I in both validation set and metacluster I (Fig. 6d). Replication of other immune infiltration findings turned out to be more challenging—tumor purity and CD4^+^ lymphocytes infiltration were significantly higher in our and metacluster I samples, but was not in chordoma I from validation set, which may be due to insufficient number of observations (Additional File 5: Figure S5d-e).

## Discussion

Our study aimed to determine DNA methylation profile in skull base chordomas as well as to investigate their possible methylation-based subclassification and the role of epigenetic abnormalities in their pathogenesis. The results indicate that there are two molecular subtypes of these tumors. These subtypes have distinct DNA methylation profile reflected by differences in global methylation level and several differentially methylated regions. In line with our findings, two epigenetic – DNA methylation-based subtypes of chordomas were also identified in the two recently published studies [13, 14]. To the best of our knowledge, this is the first report to characterize these chordoma subtypes by comparison with DNA methylation patterns obtained in NP which served as a normal controls for the study purposes. When analyzing global methylation pattern, we found chordoma I to be quite similar to the control samples with comparable overall DNA methylation level and hypermethylation of 5’ flanking regions of the genes and CGIs. The chordoma C subtype turned out to be generally hypomethylated with an increased to normal methylation level at CGIs. This epigenetic landscape of whole genome hypomethylation with hypermethylated CGIs resembles the general DNA-methylation profile of cancer cells [42, 43]. Numerous aberrantly methylated regions were identified in both chordoma subtypes. Comparison of chordoma with NP showed DMRs in many cancer-related genes as well as in genomic cluster encoding homeobox domain genes (at *HOXD3*, *HOXD4* and *HOX11*, *HOXA9*, *HOXA10*) that play an important role in both normal differentiation and tumorigenesis [44]. Comparison of the two chordoma subtypes revealed DMRs in loci encoding genes with a known role in tumorigenesis but also in regions covering numerous genes encoding short RNAs (micro RNAs and small nucleolar RNA (snoRNAs)). Both classes of small RNA play a role in cancer but their significance in pathogenesis of chordomas is poorly recognized, especially for snoRNAs [45, 46]. Our results are the first to demonstrate epigenetic mis-regulation of small RNAs in these tumors.

RNA sequencing of the same tumor samples that were included in methylation analysis allowed for further characterization of the methylation subtypes. [13, 14] Clustering, based on gene expression did not show a clear overlap between expression and methylation-based classification of the samples. Most of chordoma samples were classified in the same gene expression cluster. No gene expression-based subclassification of skull base chordomas was also found in previous RNA-seq analyses [16, 47]. The existence of the distinct expression groups of skull base chordomas was described recently [18], however, it was not observed in our data. Correlation-based analysis of the role of DNA methylation in gene expression suggests that in chordomas a small proportion of all the genes is-controlled by DNA methylation of promoter regions. The fact that genes with promoter methylation/expression correlation are enriched in the genes that are differentially expressed in chordomas as compared to NP indicates that DNA methylation contributes to pathogenesis of these tumors. These DMRs with associated gene expression changes role, include some genes that are known to play a role in tumorigenesis, including *PIK3CD* [48], *UNC5D* [49], or *NMU* [50].

Interestingly, looking for more general relationship between promoter methylation and gene expression in chordoma samples we found brachyury gene expression to be correlated with DNA methylation. Lower promoter methylation was related to higher *TBXT* expression in chordomas that is in line with the results on the regulation of this gene during cell differentiation. Lowering brachyury expression is associated with differentiation of mesenchymal stem cells into osteoblasts and adipocytes and it is accompanied by progressive methylation of its promoter region [51]. Previous observations in chordomas suggested a role of histone rather than DNA methylation in epigenetic regulation of *TBXT* [52]. We assume that both elements of epigenetic regulation may contribute to upregulating brachyury expression in chordomas.

Transcriptomic profiling in chordomas allowed for functional analysis of the differences in gene expression between tumor clusters I and C. The results clearly demonstrated that distinct molecular processes are involved in the pathogenesis of these two tumor subtypes. Gene expression pattern of cluster I tumors indicates high immune infiltration, while cluster C tumors appear to be driven by the processes related to mitosis and cell cycle. Correspondingly, pathway analysis of genes differentially expressed between the two chordoma subtypes that are controlled by DNA methylation (as examined with correlation analysis) showed overrepresentation of genes related mainly to immune infiltration pathways. Enrichment in immune component in chordoma I was observed independently in both gene expression analysis and the analysis of the relationship between DNA methylation and expression levels. These observations were confirmed by a downstream analysis with deconvolution methods. Estimation based on both methylation arrays and RNA-seq consistently show that chordoma cluster I tumors are highly enriched in immune cells. The results of deconvolution analysis were preliminary validated by immunoassaying a set of sample against T lymphocytes. We observed that they more highly infiltrate tissue samples of tumors with higher immunoreactivity score [13, 14]. Using gene expression data, we compared the estimated immune infiltration status in chordomas with common human cancer types. This clearly highlighted the difference between subtypes and showed that chordoma C subtype generally has immune scores below those observed in other caners (Fig. 4d) and can be therefore called *immune-cold*. Comparable results were observed in studies by Huo et al*.* and Zuccato et al*.* who reported that one of DNA methylation chordoma subtypes has notably higher content of immune cells [13, 14]. However, our comparative results indicate that those clusters cannot be used interchangeably.

We used data generated with EPIC microarrays for DNA copy number analysis. It showed that, in contrast to chordoma I, chordoma C subtype is notably affected by DNA copy number changes with deletion of 9p chromosomal arm as most common aberration found in nearly all tumors in this methylation cluster. This chromosomal arm contains *CDKN2A* and *CDKN2B* genes—crucial cyclin-dependent kinase inhibitors. Loss of these key cell cycle suppressors in C-type chordomas clearly corresponds to results of gene set enrichment analysis which revealed the role of cell proliferation in the pathogenesis of this chordoma subtype. We also observed notably lower expression of both genes in samples with 9p loss accordingly to previous results including a study on 384 chordoma samples that showed loss of P16/INK4A protein expression in chordomas with 9p deletion [53]. Our result showing the occurrence of 9p deletion in nearly all tumors of C subtype slightly differs from the observation by Huo et al*.* and Zuccato et al*.* [13, 14]. Both groups also showed that CNAs are more frequent in one of methylation subtypes of chordoma, but they identified 9p loss only in a minor proportion of specimens. However, Huo et al. reported a frequent loss of chromosome arm 19p (encoding cyclin E gene) in immune enriched subtype of chordomas that was found neither in our results nor in those published by Zuccato et al*.* Some differences in molecular features of the tumors as well as slightly different abundance proportions between two subtypes that are observed in ours and previously reported studies may by caused by populational differences or distinct demographic/clinical profile of each of the patient groups. Previous studies included Canadian/French [13] and Chinese [14] patients while our cohort was composed of Polish patients only. Of note, the average patients’ age in study by Huo et al. is notably lower than in our research (36.8 vs 54.28 years, respectively). Additionally, in our study we included recurrent chordomas (31%). It is not clear what was the proportion of recurrent tumors in Canadian/French and Chinese studies patient groups but *CDKNA2* deletions were reported more common in patients with higher age (age > 40 years) and in recurrent tumors [53]. Interestingly, the frequency of *CDKN2A* deletions in independent chordoma dataset (GSE140686) reported by Huo et al*.* [14] was higher than in our study group (34.8% vs 28.12%, respectively).

A notable effort in chordoma research is focused on identification of prognostic factors. DNA methylation-based subclassification of skull base chordomas showed a clinical relevance. Both the results by Zuccato et al*.* and Huo et al*.* [13, 14]. indicated prognostic role of immune component in DNA methylation pattern, but the authors found dissimilar results. Observations by Zuccato et al*.* indicate shorter overall survival (OS) in patients with immune-hot chordoma subtype identified in DNA methylation-based clustering. Contrastingly, Huo et al*.* [14] reported worse outcome in patients with low immune cell content and higher tumor purity [13, 14]. We did not find a significant difference between the two DNA methylation-based subtypes of chordomas in terms of patients’ overall survival. The molecular features that we found as discriminating epigenetic chordoma subtypes are known as relevant prognostic factors in other studies. Previously, a higher immune infiltration status determined by microscopic assessment of CD3 + and CD8 + cells count was identified as related to better prognosis in patients with spinal chordomas [54, 55] but another study showed that higher content of CD8-positive cells is related to shorter survival [56].

In previous studies, DNA copy number status in skull base chordomas also showed prognostic value. Deletion of 9p21, that we observed in nearly all subtype C chordomas was associated with worse recurrence-free survival but not OS in study by Bai et al*.* [10]. The same study also showed a prognostic relevance of 22q (encoding *SMARCB1* gene) deletions [10]. Changes in 22q region were not observed in our patients’ group probably because it is more homogeneous and contained only one histological subtype of classical chordomas. Clinical relevance of *CDKN2A* expression and deletion of this gene locus were specifically address in the other study on classical and chondroid chordomas [40]. It revealed *CDKN2A* deletion in approximately 50% of the tumors but significant relationship between DNA copy number and patients’ survival was not observed [40].

Our results of copy number analysis showed prognostic value of chromosomal instability. We did not find survival difference between patients with 9p (*CDKN2A* locus) loss and 9p stable patients, but we observed a significant relationship of the higher level of copy number changes in tumors with shorter survival as clearly illustrated by significant difference in OS between patients with high and low number of CNAs. This observation is concordant with a general finding that chromosomal instability in human cancer is a biomarker of poor prognosis [57].

Besides a potential prognostic value, the observed biological difference between two chordoma subtypes with potentially important clinical implications in terms of the therapy. Immunotherapy, that include the use of immune checkpoint inhibitors (ICI) is a promising therapeutic avenue in chordoma and number of clinical trials on this field has been already initiated [58]. Importantly, the level of tumor-infiltrating lymphocytes is generally one of most relevant indicators of response to this therapy [59]. Therefore, this treatment may potentially be more beneficial in patients with chordoma I tumors. In turn, the role of cell cycle regulation and deletion of *CDKN2A* in chordoma C suggests an efficiency of cell cycle targeting therapy in these patients. Inhibitor of cyclin-dependent kinases 4/6, palbociclib showed an inhibitory activity in chordoma cell lines with P16/INK4A loss [60] and more recently in 1 (out of 2) chordoma xenografts with this 9p deletion [61]. The synergistic effect of combination of rapamycin and palbociclib in chordoma cell lines with PTEN and P16/INK4A loss was also shown [62]. Chordoma C patients harboring 9p are those who could potentially benefit from this type of treatment. The clinical trial on efficiency of palbociclib in chordoma patients has already been initiated. Its protocol includes molecular tumor profiling that hopefully would provide the rationale for this hypothesis [63]. Our findings are first to indicate that immune infiltration is negatively correlated with *CDKNA2* loss and therefore immunotherapy and cell-cycle inhibitors may have different target subpopulations, among patients, affected by chordoma.

## Conclusions

Two distinct chordoma subtypes (subtype C and I) with different patterns of aberrant DNA methylation have been identified upon genome-wide DNA methylation analysis. They have different profiles of both global and locus-specific methylation pattern as well as distinct gene expression. Differences in gene expression indicate immune activation in I chordomas and enhanced cell proliferation in C chordomas. Immune enrichment in I chordomas found in transcriptomic profiling is also confirmed by results of analysis with deconvolution methods. C chordomas are characterized by higher chromosomal instability, according to results of copy number analysis and they have 9p deletion causing downregulation of cell cycle inhibitors *CDKN2A/B*. Tumor subtypes do not differ significantly in terms of prognosis, however, there is a significant influence of chromosomal instability level on shorter survival.

## Supplementary Information

**Additional file 1.** Entanglement plot of the methylation and RNA sequencing-based clustering. Both methods used (side1side and side2side) have entanglement of 0.19 and 0.10 respectively, which indicate some relation between both clustering methods, despite global differences.

**Additional file 2.** WGCNA diagnostics - first four panels (Scale Free Topology Model Fit, Max Connectivity, Mean Connectivity, and Median Connectivity) all point to selection of power = 10. Bottom 4 panels show clustering of the modules.

**Additional file 3.** GSEAlm estimate of gene expression across chromosomal bands in the whole genome.

**Additional file 4.** The results of immunohistochemical staining of selected chordoma C and chordoma I samples with antibodies against CD3, CD4 and CD8.

**Additional file 5.** Accompanying Figure 6, presents the remainder of the analysis; **a**) fraction of methylation of probes methylated, split by relation to gene **b**) Uniform Manifold Approximation and Projection of all samples with original clusters and metaclusters **c**) methylation of probe in the brachyury (T ot TBXT) promoter proximity, confirming hypomethylation of brachyury promoter also in the validation set **d**) MethylResolver estimate of tumor purity (inverse of fraction of stromal cells) in samples **e**) MethylResolver estimate of samples infiltration by helper lymphocytes T.

**Additional file 6.** Interactive file, depicting all differentially methylated regions (DMRs).

**Additional file 7.** Supplementary tables: DMP - differentially methylated probes, DMR - differentially methylated regions, DEG - differentially expressed genes, GSEA - gene set enrichment analysis, MethExpr_Correlation - correlation of promoter methylation with gene expression, DEG_DMR_Correlation - correlation of differentially expressed genes with methylation in DMRs, DEG_DMR overrepresentation - Overrepresentation analysis of DEGs correlated with DMRs, WGCNA_modules_significance - statistical testing of WGCNA modules association with methylation clusters, Immune_deconvolution - statistical testing of immune cell deconvolution with methylation clusters, conumee - estimation of CNAs in chordoma samples, GSEAlm - GSEAlm estimate of gene expression across chromosomal bands in the whole genome.

## Data Availability

The datasets generated with RNAseq and EPIC arrays aare available at Gene Expression Omnibus repository, GSE230168 (https://www.ncbi.nlm.nih.gov/geo/query/acc.cgi?acc=GSE230168).

## Abbreviations

5’UTR: 5’ Untranslated region
Bp: Base pairs
CGI: CpG island
CNAs: Copy number variations
DEG: Differentially expressed gene
DMP: Differentially methylated probe
DMR: Differentially methylated region
GO: Gene Ontology
GSEA: Gene set enrichment analysis
NP: Nucleus pulposus
RNA-seq: RNA sequencing
snoRNAs: Small nucleolar RNA
SNP: Single nucleotide polymorphism
TSS: Transcription start site
WHO: World Health Organization
WGCNA: Weighted correlation network analysis
