# Supplementary figures and images for: DNA methylation, combined with RNA sequencing, provide novel insight into molecular classification of chordomas and their microenvironment
Source: Acta Neuropathol Commun. 2023 Jul 11;11:113. doi: 10.1186/s40478-023-01610-0 (PMC10337070; doi:10.1186/s40478-023-01610-0)

## Expression

## Methylation

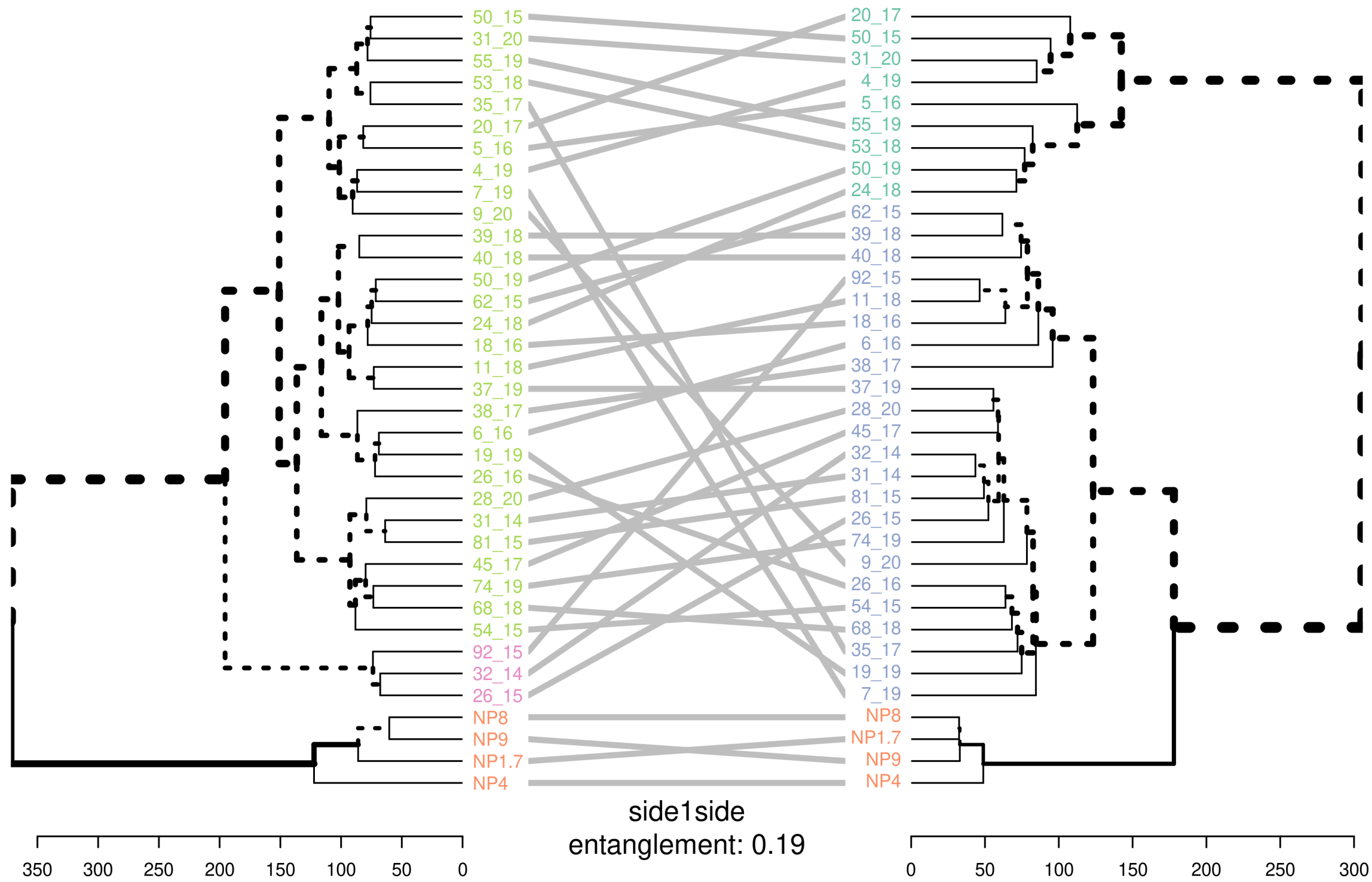

## Expression

## Methylation

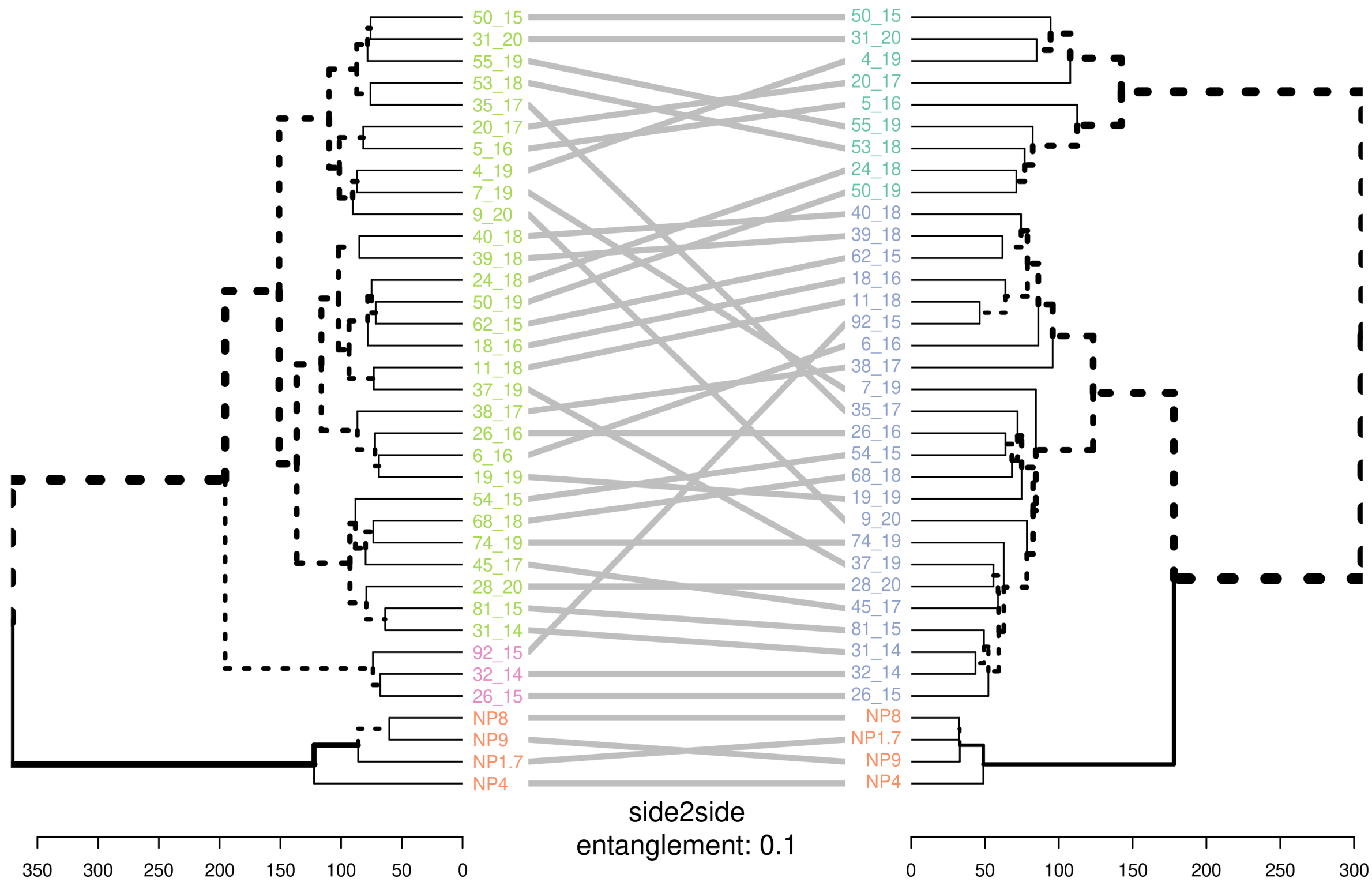

Supplement: Supplementary file 1 — Additional file 1. Entanglement plot of the methylation and RNA sequencing-based clustering. Both methods used (side1side and side2side) have entanglement of 0.19 and 0.10 respectively, which indicate some relation between both clustering methods, despite global differences. [file 40478_2023_1610_MOESM1_ESM.pdf]

WGCNA diagnostics

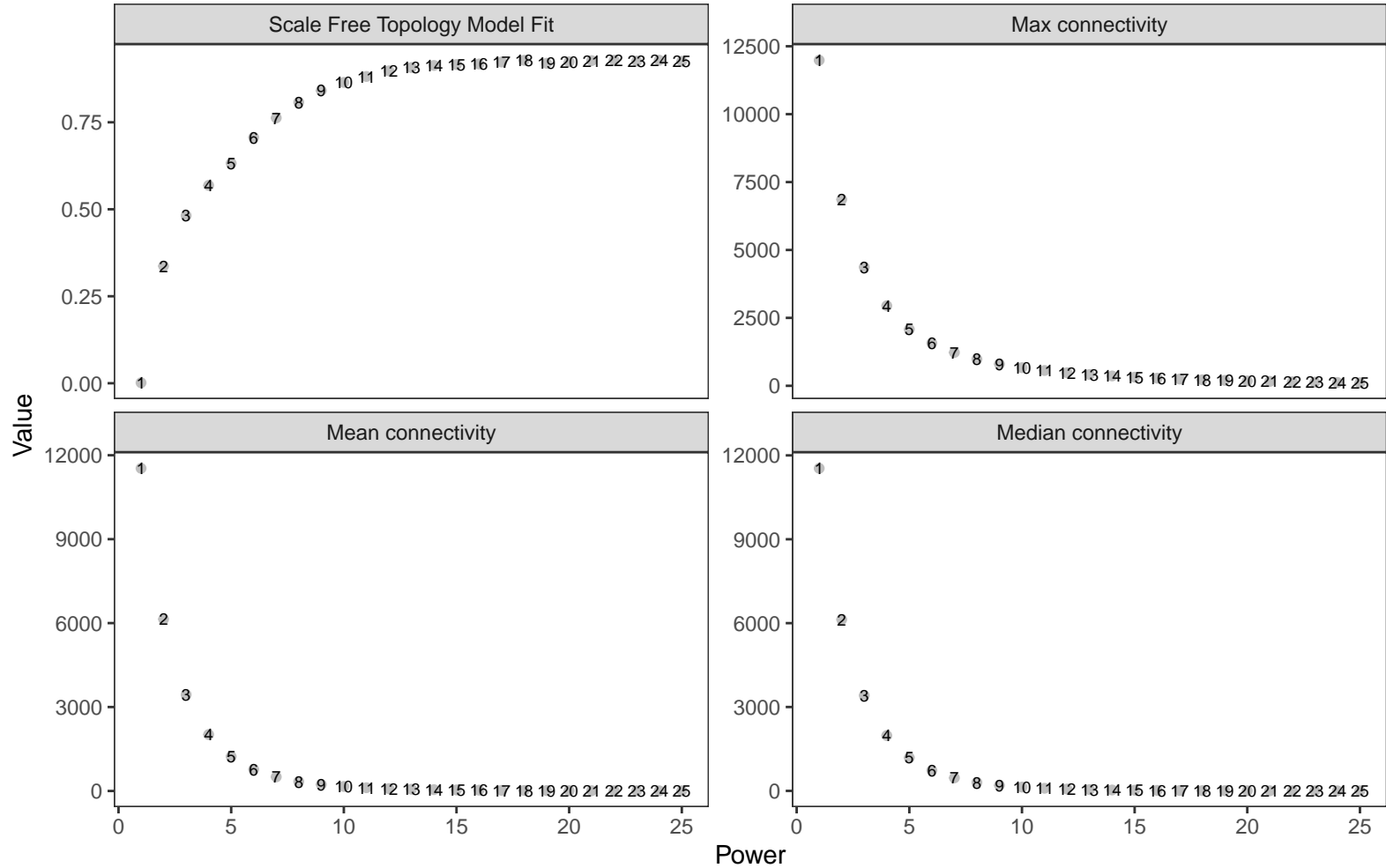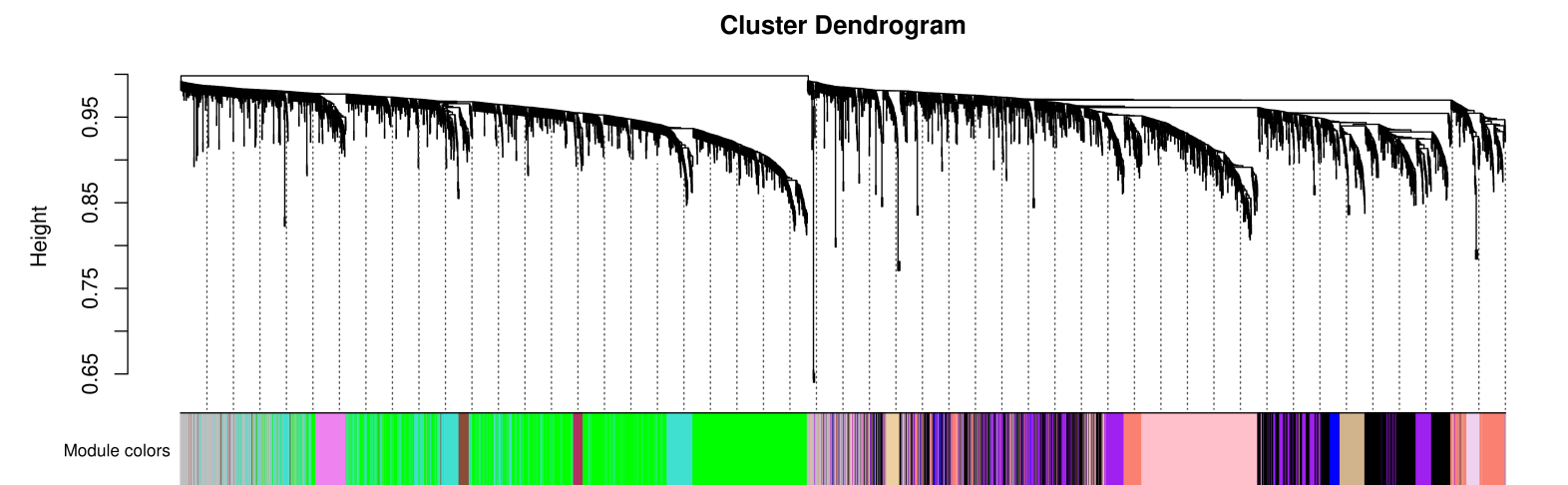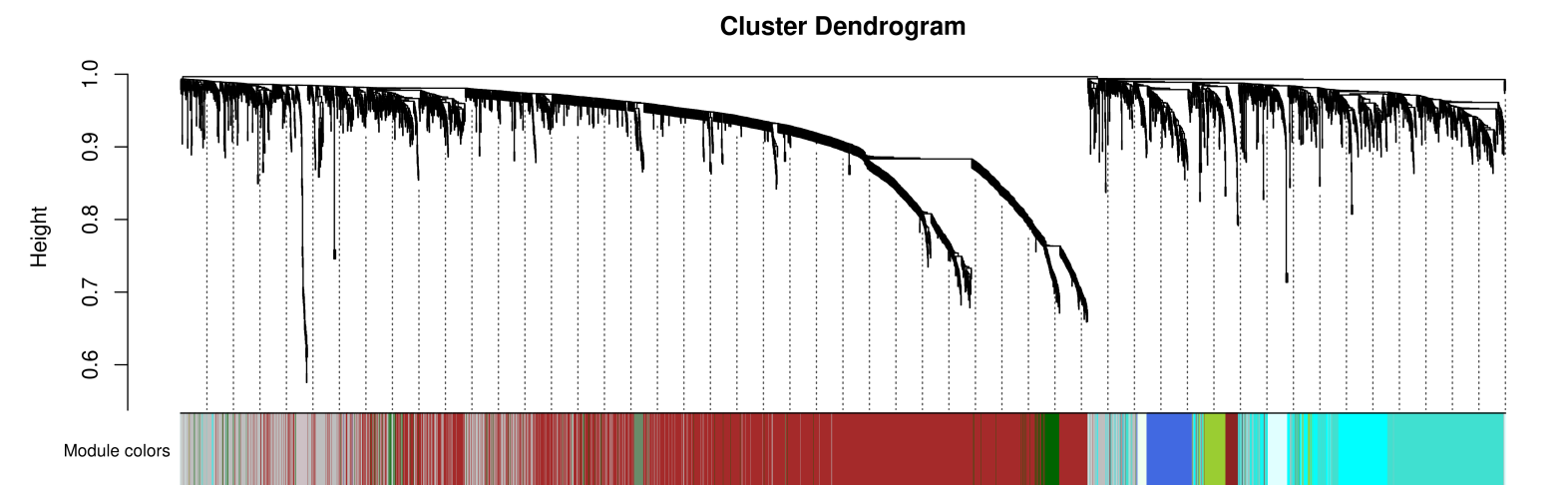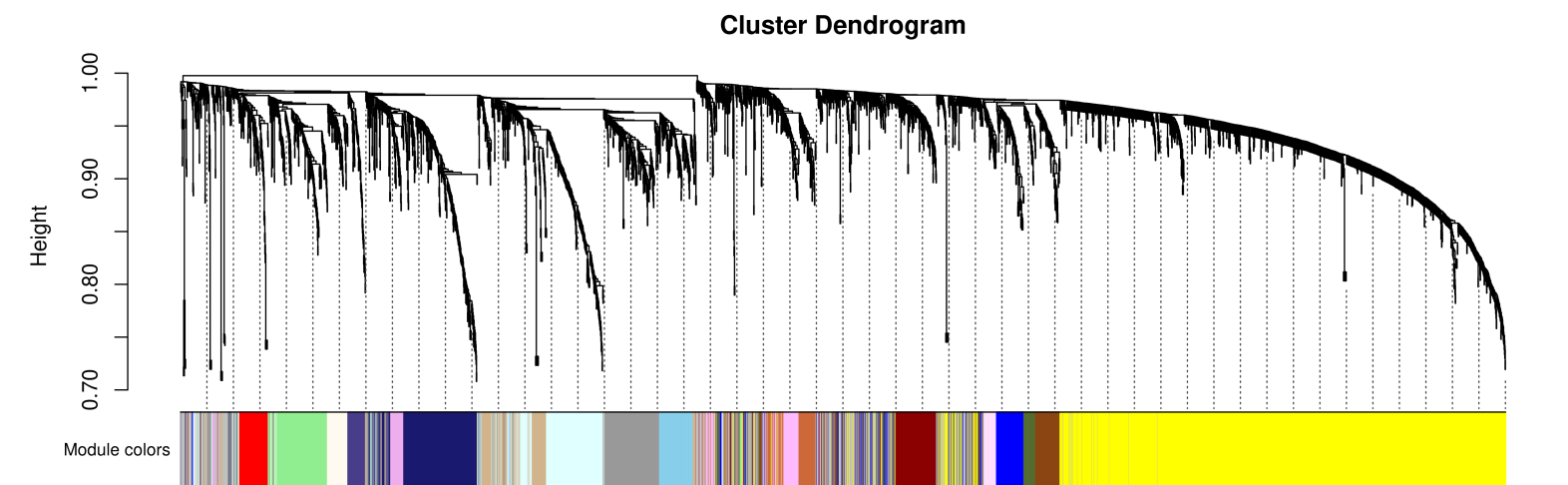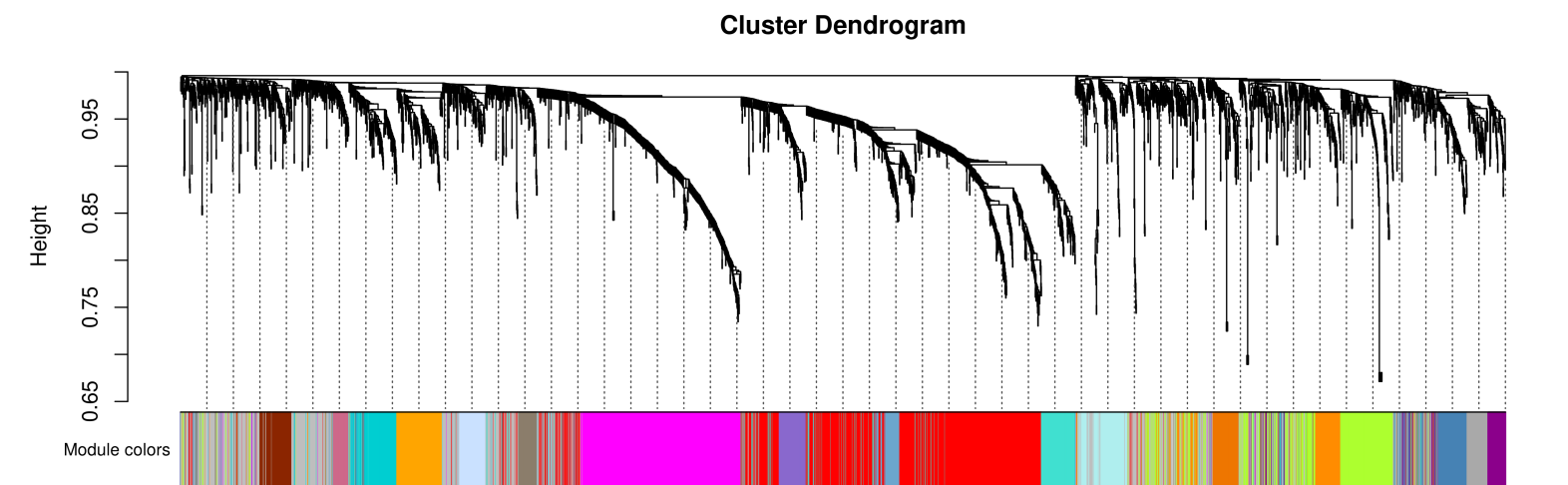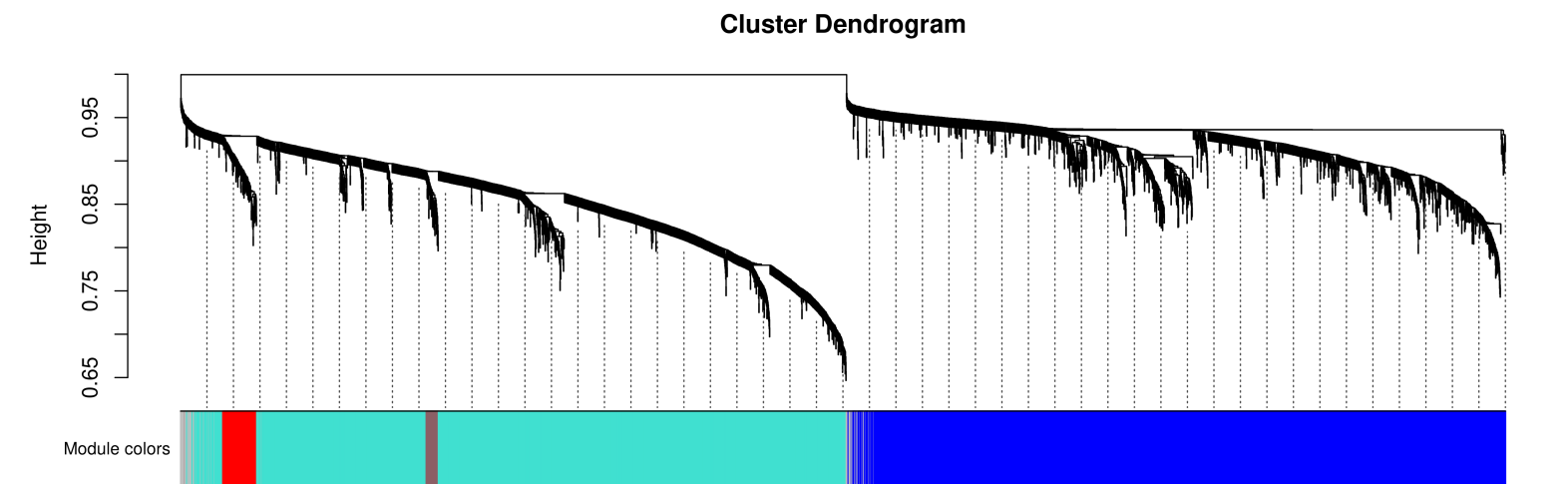

Supplement: Supplementary file 2 — Additional file 2. WGCNA diagnostics - first four panels (Scale Free Topology Model Fit, Max Connectivity, Mean Connectivity, and Median Connectivity) all point to selection of power = 10. Bottom 4 panels show clustering of the modules. [file 40478_2023_1610_MOESM2_ESM.pdf]

GSEAIM normalization of expression across chromosomal bands

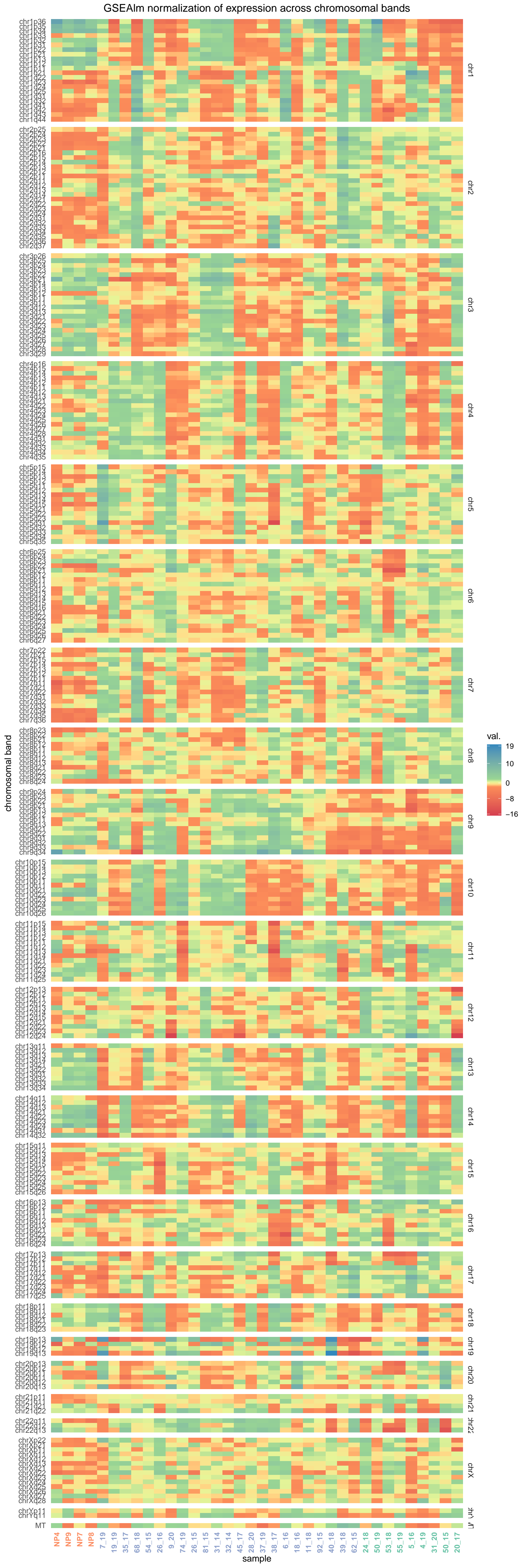

Supplement: Supplementary file 3 — Additional file 3. GSEAlm estimate of gene expression across chromosomal bands in the whole genome. [file 40478_2023_1610_MOESM3_ESM.pdf]

# Chordoma I

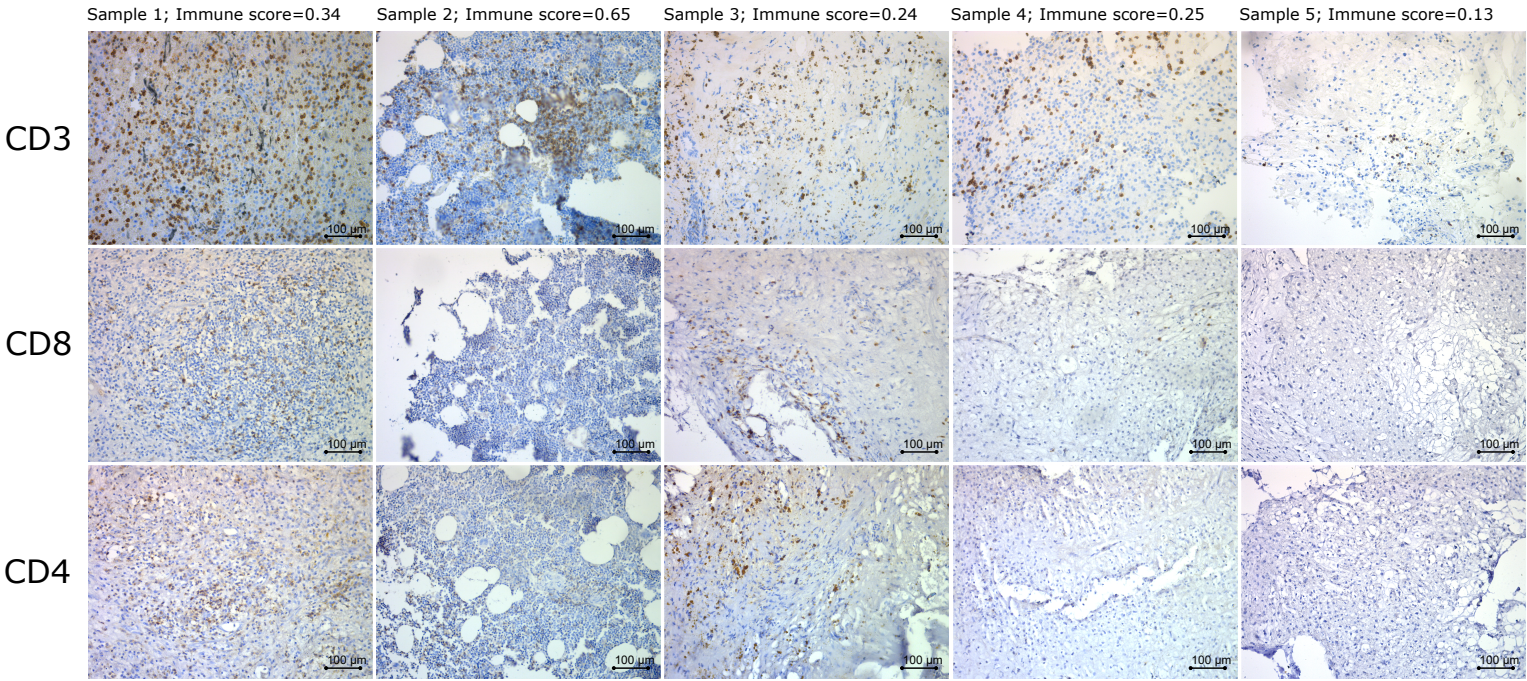

# Chordoma C

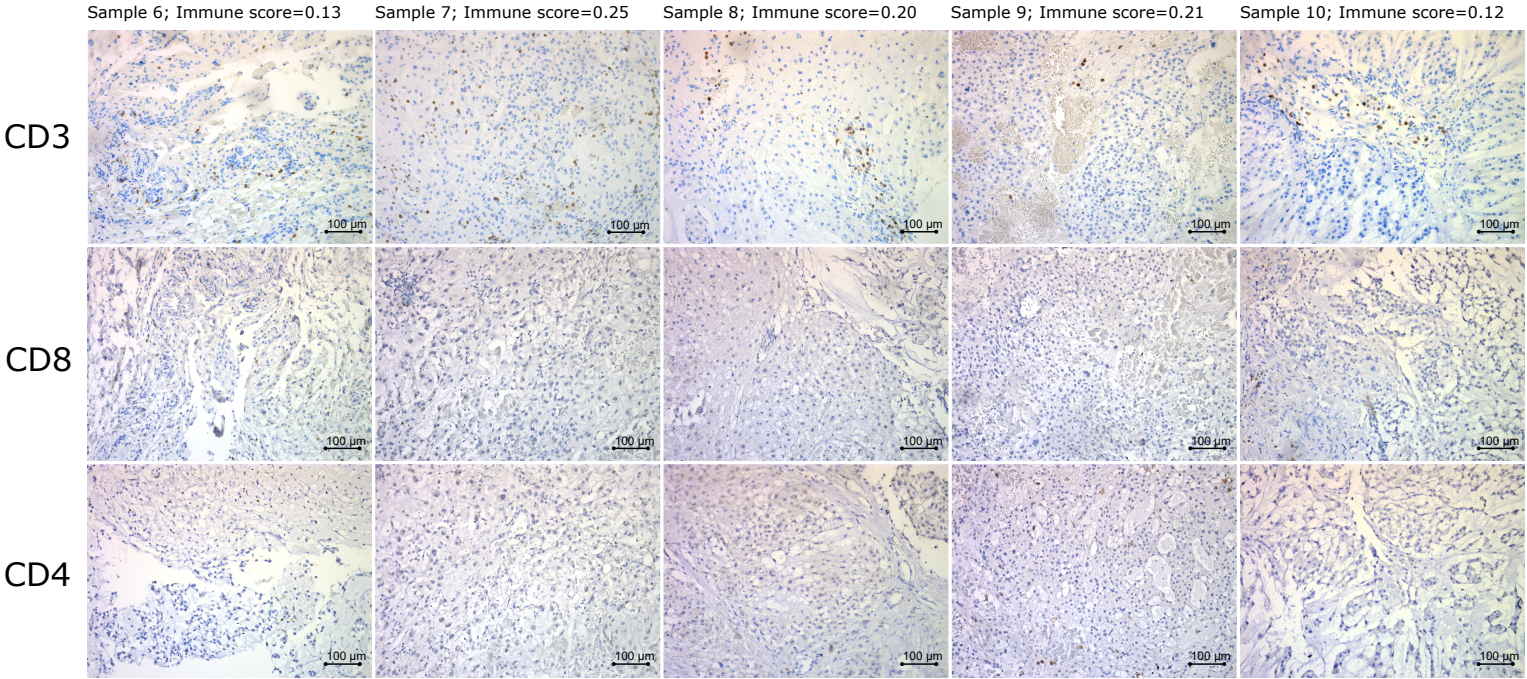

Supplement: Supplementary file 4 — Additional file 4. The results of immunohistochemical staining of selected chordoma C and chordoma I samples with antibodies against CD3, CD4 and CD8. [file 40478_2023_1610_MOESM4_ESM.pdf]

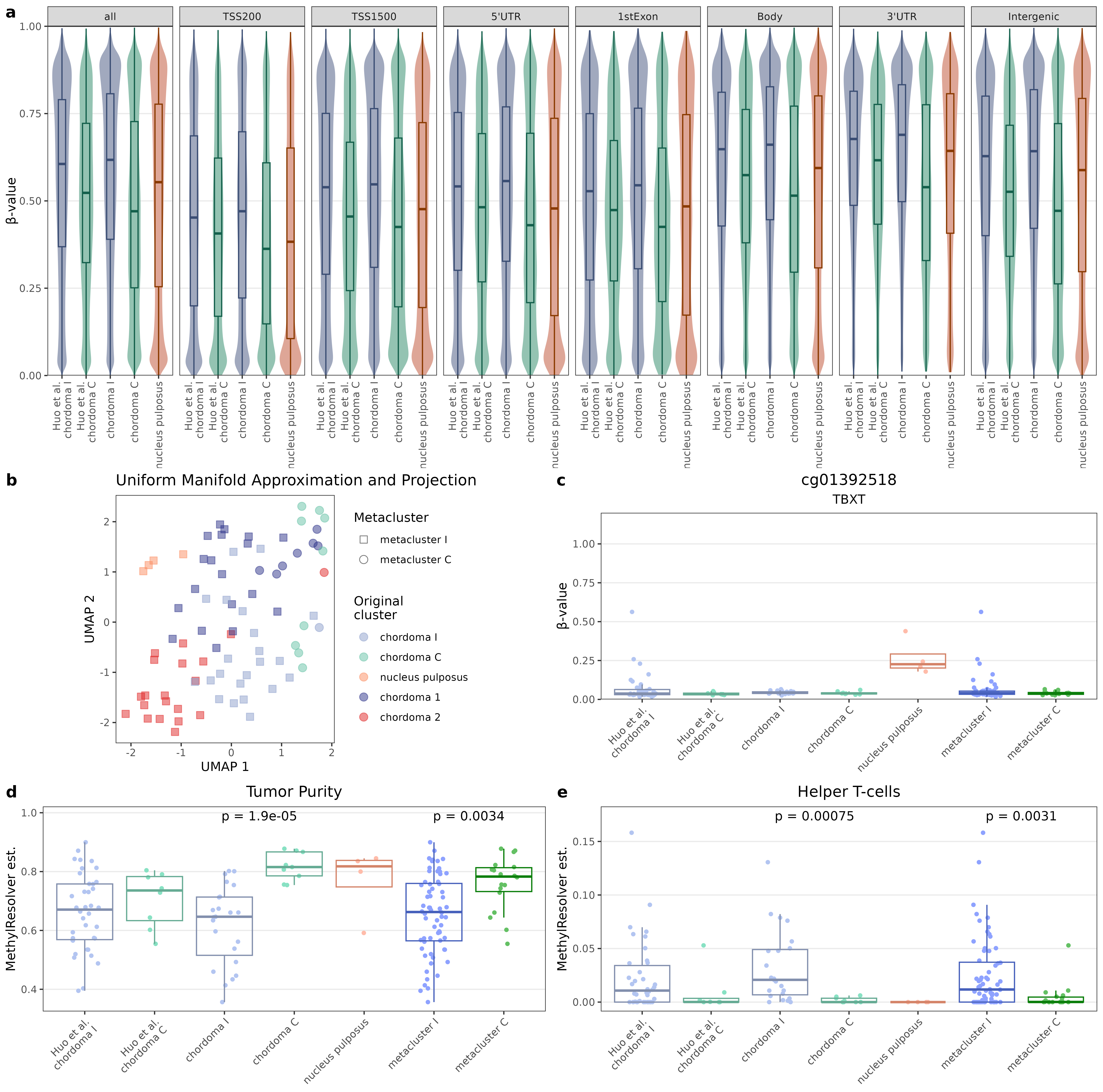

Supplement: Supplementary file 5 — Additional file 5. Accompanying Figure 6, presents the remainder of the analysis; a) fraction of methylation of probes methylated, split by relation to gene b) Uniform Manifold Approximation and Projection of all samples with original clusters and metaclusters c) methylation of probe in the brachyury (T ot TBXT) promoter proximity, confirming hypomethylation of brachyury promoter also in the validation set d) MethylResolver estimate of tumor purity (inverse of fraction of stromal cells) in samples e) MethylResolver estimate of samples infiltration by helper lymphocytes T. [file 40478_2023_1610_MOESM5_ESM.png]
